# Pancancer analysis of the correlations of HS6ST2 with prognosis, tumor immunity, and drug resistance

**DOI:** 10.1038/s41598-023-46525-x

**Published:** 2023-11-06

**Authors:** Weiwei Chen, Xia Li, Youqin Jiang, Daguang Ni, Longfei Yang, Jixiang Wu, Mingcheng Gao, Jin Wang, Jianxiang Song, Wenyu Shi

**Affiliations:** 1 Medical School of Nantong University, Nantong, 226007 China; 2 Department of Oncology, Affiliated Hospital of Nantong University, Nantong, 226001 China; 3 Department of Radiotherapy, The Sixth Affiliated Hospital of Nantong University, Yancheng Third People’s Hospital, The Yancheng School of Clinical Medicine of Nanjing Medical University, Yancheng, 224002 China; 4 Department of Cardiothoracic Surgery, The Sixth Affiliated Hospital of Nantong University, Yancheng Third People’s Hospital, The Yancheng School of Clinical Medicine of Nanjing Medical University, Yancheng, 224002 China; 5 Department of General Medicine, The Sixth Affiliated Hospital of Nantong University, Yancheng Third People’s Hospital, The Yancheng School of Clinical Medicine of Nanjing Medical University, Yancheng, 224002 China

**Keywords:** Biotechnology, Cancer, Computational biology and bioinformatics, Genetics

## Abstract

HS6ST2 has ability to encodes a member of the heparan sulfate (HS) sulfotransferase gene family, which catalyze the transfer of sulfate to HS and a crucial regulator of cell growth, differentiation, adhesion, and migration. Although mounting evidence supports a vital role for HS6ST2 in tumorigenesis of some cancers, no pan-cancer analysis of HS6ST2 has been reported. Therefore, we aimed to explore the prognostic value of HS6ST2 in 33 cancer types and investigate its potential immune function. Based on data from The Cancer Genome Atlas, Cancer Cell Lines Encyclopedia, Genotype Tissue Expression, and GSCA, we used a range of bioinformatics approaches to explore the potential carcinogenic role of HS6ST2, analysis of HS6ST2 and prognosis, DNA methylation, RNA methylation, microsatellite instability (MSI), tumor mutation burden (TMB), and immune cell infiltration in different tumors. The results show that HS6ST2 was highly expressed in most cancers but lower in Breast invasive carcinoma, Kidney Chromophobe, Kidney renal clear cell carcinoma, Kidney renal papillary cell carcinoma, and Uterine Corpus Endometrial Carcinoma. Moreover, HS6ST2 is positively or negatively associated with prognosis in different cancers. HS6ST2 expression was not only associated with MSI in 5 cancer types and associated with TMB in 10 cancer types, and it's significantly correlated with DNA methylation in 13 types of cancer, but it's correlated with RNA methylation related genes in most cancer. HS6ST2 expression was correlated with immune cell infiltration, immune-related genes, tumor immune microenvironment, and drug resistance in various cancers. Eventually, HS6ST2 was validated in human lung adenocarcinoma tissues. Our study reveals that HS6ST2 can function as a prognostic marker in various malignant tumors because of its role in tumorigenesis and tumor immunity.

## Introduction

Cancer was one of the primary reasons of death in 2020 and was responsible for nearly 10 million deaths globally^1^. Immunotherapy has recently emerged as the most promising modern cancer treatment and includes surgery, radiation therapy, gene therapy, and chemotherapy^2^. Immunotherapy treatments that target PD-1/PD-L1 or CTLA4 are some of the most successful tumor immunotherapies and have significantly enhanced the effectiveness of cancer treatment^3^. Nevertheless, cancer cells also have clever strategies to avoid immune system attack; for example, HLA deletion caused by β2MG mutation leads to a lack of presentation of neoantigens on the cell surface, limiting the response of cytotoxic T cells and ultimately leading to the development of resistance to PD-1 treatment^4^. Moreover, the economic burden of cancer on nations throughout the world is increasing^5^. Therefore, the discovery of novel cancer biomarkers and therapeutic targets is urgently required.

The extracellular matrix, basement membrane, and cell surface all include heparan sulfate proteoglycans (HSPGs), which interact with different ligands to regulate cell proliferation, differentiation, migration, and adhesion^6^. Heparan sulfate 6-O-sulfotransferase 2 (HS6ST2) belongs to the heparan sulfate (HS) sulfotransferase family and is responsible for catalyzing the transfer of sulfate to HS^7^. HS6ST2 is overexpressed in the bronchial epithelial cells of human idiopathic pulmonary fibrosis, and methylation of the CpG island methylation site of this gene (cg19782749) is related to susceptibility to SARS-CoV-2 infection^8,9^. Downregulation of HS6ST2 can increase MMP13 protein levels and promote osteoarthritis development by regulating the activity of p38 MAPK, and these effects can be reversed by overexpression of miR-23b-3p^10^. Numerous investigations have shown that HS6ST2 contributes to the development of tumors. For example, inhibition of HS6ST2 impacts IL-8 and FGF2 expression in ovarian cancer cells and reduces tumor growth in vivo^11^. Another study illustrated that HS6ST2 was highly expressed in papillary thyroid cancer, thyroid cancer, and high-grade cartilage tumor tissues^12^, though it was expressed at low levels in glioma tissues^13^, and HS6ST2 was also found to promote the development of thyroid cancer^14,15^. HS6ST2 expression was found to be negatively related to OS and was an independent prognostic factor for clear cell renal cell carcinoma patient outcomes^16,17^. Furthermore, HS6ST2 can be downregulated by miR-145-5p, limiting the development of renal cell carcinoma (RCC) cells^18^. HS6ST2 also regulates the Wnt, TGF-β, and Notch signaling pathways, promoting tumor development. For instance, HS6ST2 not only is a common target of Wnt/TGF-β signaling and promotes mouse mammary and intestinal tumorigenesis^19^ but also increases TGF-β-induced IL-11 production and promotes human breast tumor growth^20^. Silencing of HS6ST2 reduces tumorigenesis and metastasis in human pancreatic cancer through Notch-mediated epithelial–mesenchymal transition (EMT)^21^. Moreover, HS6ST2 is elevated in non-small cell lung cancer (NSCLC)^22^, and HS6ST2 overexpression is related with progression of NSCLC^23^. In addition, silencing HS6ST2 decreases the viability of human lung and ovarian cell lines in the presence of a sublethal dose of Taxol^24^. However, the functional and molecular mechanisms of HS6ST2 in carcinogenesis remain to be explored.

Therefore, we downloaded the most recent information from the Genotype-Tissue Expression (GTEx), Cancer Cell Line Encyclopedia (CCLE), and The Cancer Genome Atlas (TCGA) databases to comprehensively examine the expression of HS6ST2 and its relationship with prognosis in 33 cancers. Furthermore, the association between DNA methylation, immune cell infiltration (ICI), microsatellite instability (MSI) and tumor mutation burden (TMB), and HS6ST2 expression in 33 malignancies was investigated. Coexpression analysis was used to dissect the relationships between HS6ST2 expression and the expression of genes related to RNA modification, the immune system, mismatch repair (MMR), and treatment response in these malignancies. The biological function of HS6ST2 in cancers was examined by employing gene set enrichment analysis (GSEA), and the results were verified with in vitro experiments with lung adenocarcinoma (LUAD) cell lines. HS6ST2 was found to have a critical role in cancer immunity by modulating TMB, MSI status, and tumor-infiltrating immune cell levels and being a predictive factor across malignancy types.

## Methods

### Data collection

The gene expression RNAseq and somatic mutation data of 33 malignant tumors with the starting title of GDC TCGA were downloaded from the DATA SETS of the UCSC Xena database (https://xenabrowser.net/datapages/). We also retrieved gene expression RNAseq of 26 healthy human tissue, which corresponds with the tumor tissues of the TCGA database, in the Genotype-tissue expression (GTEx) database, from the UCSC Xena database, except for muscle, adipose, heart, nerve, pituitary, salivary gland, small intestine, spleen, blood vessel, and vagina. We obtained a titled Expression Public 22Q1 dataset, which contains HS6ST2 RNA expression in Thirty-three types of cancer cell line expression, from the CCLE database (https://portals.broadinstitute.org/ccle/).

### HS6ST2 methylation profiles of cancers in GSCA

The GSCA database was accessed to investigate the relationship between HS6ST2 methylation and HS6ST2 mRNA expression between various tumor types and paracancerous tissues.

### Correlation of HS6ST2 expression with RNA modifications-related genes

The expression levels of HS6ST2 were compared to those of genes involved in m6A, m5C, and m1A modifications in various malignancies using data from TCGA.

### Analysis of the relationship between prognosis and HS6ST2

TCGA patient outcome data were analyzed using Kaplan‒Meier (KM) and univariate Cox regression analyses to determine disease-free interval (DFI), disease-specific survival (DSS), overall survival (OS), and progression-free interval (PFI)^25^. To analyze the correlation between HS6ST2 expression and survival in various malignancies, we utilized the R packages "survival" and "forestplot" to conduct univariate Cox regression analysis.

### Correlation of MMR gene expression, MSI status and TMB level with HS6ST2

We quantified the expression levels of MMR genes in various malignancies base upon TCGA expression data and identified the interrelationships of these MMR genes with HS6ST2. We calculated MSI and TMB scores with a Perl script utilizing information on somatic mutations and the VarScan2 variant detector from the UCSC Xena database for tissue samples representing 33 different cancer types. Spearman's rank correlation coefficient was used to examine the relationship of HS6ST2 expression with MSI status and TMB^26^.

### The connection between immunity and HS6ST2 expression

The ESTIMATE algorithm was used to find out the connection between the tumor microenvironment (TME) and HS6ST2 expression. The R packages "limma" and "estimate" were performed to analyze the correlation between HS6ST2 expression and immune and stromal scores to figure out the relationship of HS6ST2 with immune infiltration^27^. We utilized CIBERSORT to calculate TCGA ICI scores (https://cibersortx.stanford.edu/) and to determine relative scores of 22 different types of immune cells in 33 malignancies. The correlation of the level of each tumor-infiltrating immune cell with HS6ST2 expression was uncovered via Spearman's rank correlation analysis. Furthermore, we applied the R package "limma" to investigate the expression of genes involved in immunity correlates with HS6ST2 across 33 distinct cancer types, and we visualized our findings with the "reshape2" and "RColorBrewer" packages.

### Drug sensitivity analysis

The GSCA database has been utilized to determine the relationship between drug sensitivity and HS6ST2 mRNA expression.

### GSEA

GSEA was utilized to examine the biological function of HS6ST2 in cancers. Gene sets from Kyoto Encyclopedia of Genes and Genomes (KEGG) and Gene Ontology (GO) were retrieved from the GSEA database^28^. The R package "Cluster Profiler" was used to conduct both studies.

### Clinical samples

Fifty matched samples of LUAD and surrounding normal tissues were obtained at the Third People's Hospital in Yancheng. Among them, there were 32 males and 28 females, none of whom had received radiation or chemotherapy. Out of the total, 24 were smokers. The diagnosis was confirmed using histopathological methods. There were 19 patients in stage I, 15 patients in stage II, 12 patients in stage III, and 4 patients in stage IV. After the tissues were surgically removed or obtained through bronchoscopy, the specimens were frozen in a refrigerator at − 80 °C for future research. All fifty patients provided written informed consent, This project was approved by the Ethics Committee of the Sixth Affiliated Hospital of Nantong University (Approval Number: LS20150413). All experiments were conducted in accordance with the Helsinki Declaration, and informed consent was obtained from all participants.

### Reverse transcription-polymerase chain reaction (RT‒qPCR)

We extracted RNA from fresh tissue samples using the Trizol method. The isolated RNA was then reverse transcribed into cDNA using a reverse transcription kit provided by a Chinese genetic engineering company. The PCR reaction mixture was placed in a thermal cycler and subjected to specific temperature and time conditions for PCR amplification. The process involved an initial denaturation step to separate the DNA strands, an annealing step to allow the primers to specifically bind to the template DNA, and an extension step for the polymerase to synthesize new DNA strands. A total of 45 cycles were performed, and the maximum system volume was 20 μl. The ANanoDrop3000 (Thermo Fisher Scientific, Waltham, USA) was used to quantify fluorescence expression. The relative expression level of HS6ST2 was determined using the 2−ΔΔCT method. The primer sequences used were as follows: β-actin FORWARD: CTGGCACCACACCTTCTACAATG, REVERSE: TGGGTCATCTTCTCACGGTTGG. HS6ST2 FORWARD: GCTGCTACACTGGCGATGACTG, REVERSE: CCTGGCGGTTGTTGGCTAGATTG.

### Immunohistochemistry (IHC) staining analysis

Immunohistochemistry (IHC) is a method that utilizes specific antibodies to bind to proteins in tissues for detecting the expression of target proteins. In our study, we validated the expression of HS6ST2 in LUAD tissue and corresponding adjacent non-cancerous tissue samples. The tissues were fixed using formalin to preserve their morphology and structure. Antigen retrieval was achieved through heat-induced epitope retrieval. Blocking of nonspecific binding sites was performed using 10% goat serum to reduce false-positive results. The primary antibody against HS6ST2 (Abcam, 1:200) was added to the samples and incubated overnight at 4 degrees Celsius. After incubation, the samples were washed three times with PBS for 5 min each to remove unbound antibodies and other nonspecific substances. Subsequently, a secondary antibody labeled with horseradish peroxidase (HRP) (Beyotime, 1:100) was added and incubated for 30 min. The samples were washed again with PBS four to eight times for 5 min each to remove unbound secondary antibodies and other nonspecific substances. DAB (3,3'-diaminobenzidine) chromogen was added and allowed to react for 5–15 min. The development of a yellow color was observed under a microscope, and the reaction was immediately terminated. Finally, the tissue sections were examined under a microscope to evaluate the expression and localization of the target protein.

### Western Blot analysis

We placed the fresh tissue specimens in liquid nitrogen for preservation. The tissue samples were then taken out and added to RIPA lysis buffer (RIP). The tissue samples were completely homogenized using a handheld homogenizer and subjected to ice-cold lysis for 30 min. After that, the samples were centrifuged at 12,000×*g* for 15 min at 4 °C using a centrifuge. The supernatant was collected, and the protein concentration was measured using a BCA protein assay kit. The samples were heated at 95 °C–100 °C for 5 min to denature the proteins. 7.5% polyacrylamide gel was prepared, and the samples, including molecular weight markers, were loaded into the gel wells. The gel was placed into an electrophoresis chamber, and the electrophoresis buffer was added. The samples were subjected to electrophoresis at a constant voltage of 100 V until the samples migrated to the bottom of the gel. PVDF membrane was prepared and cut to match the size of the gel. The membrane was pre-soaked in methanol and then placed in a transfer apparatus. The gel was transferred onto the membrane, ensuring contact without any air bubbles. After transfer, the membrane was blocked with 5% non-fat milk powder at room temperature for 1 h to prevent non-specific binding. Following three washes with TBST for 5 min each, the membrane was incubated with the primary antibody against HS6ST2 (1:1000) β-actin (1:1000) at 4 °C for 24 h. After three washes with TBST for 5 min each, the membrane was incubated with the secondary antibody labeled with HRP (1:10,000) at room temperature for 2 h. After three washes with TBST for 5 min each, the membrane was exposed to a chemiluminescent substrate from Thermo Fisher Scientific for image development.

### Statistical analyses

Log2 transformation was used to standardize all the gene expression data. The bioinformatics data were analyzed statistically with R (Version 4.1.3). GraphPad Prism (version 8) was used to analyze the statistical significance of the in vitro data.

### Ethical approval

The Ethics Committee of Yancheng Third People's Hospital, the Sixth Affiliated Hospital of Nantong University in Jiangsu Province, China, approved this research.

## Results

### Differential expression of HS6ST2 between tumor and para-carcinoma tissues

**Figure 1:**
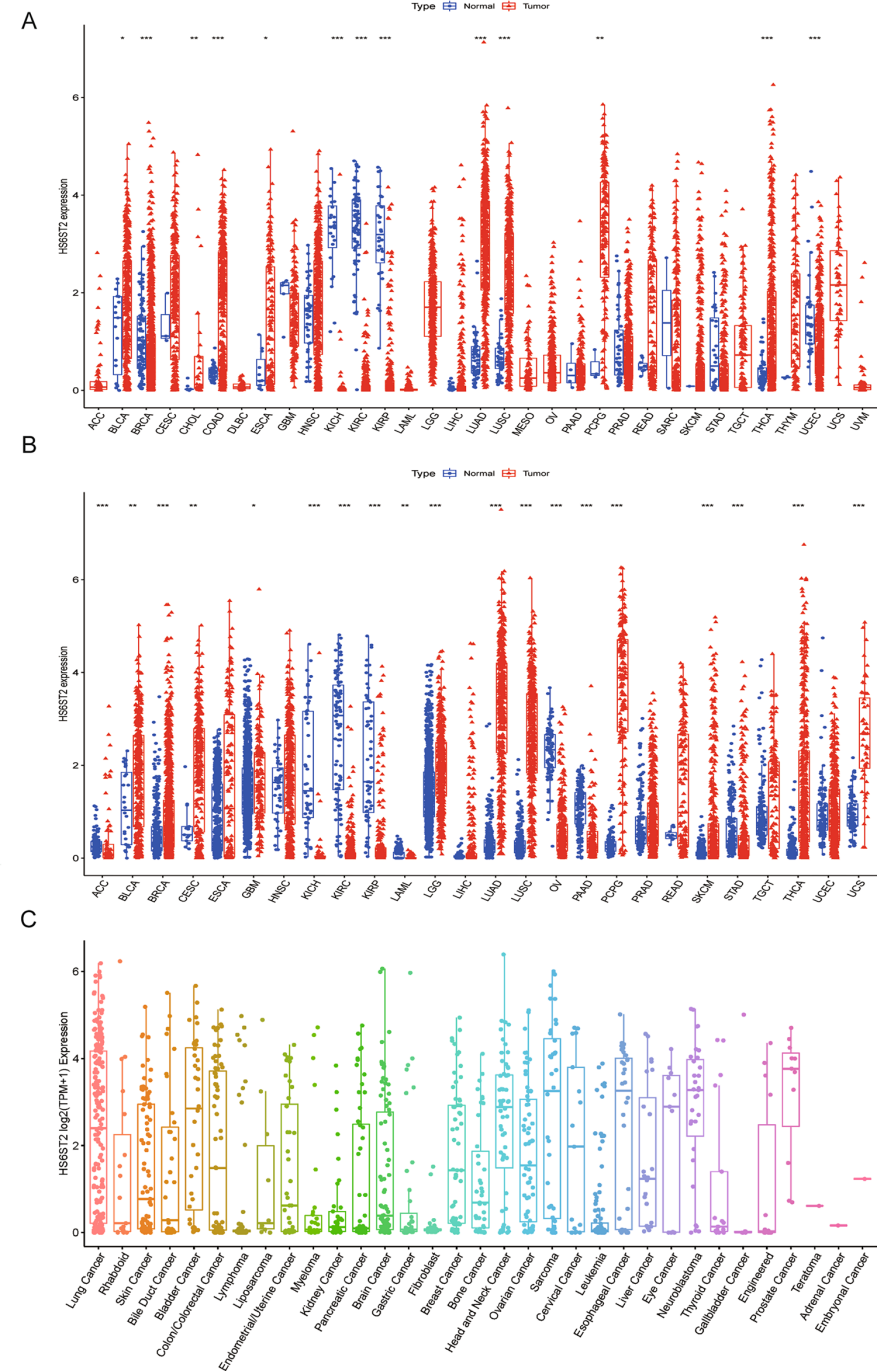
Differential expression of HS6ST2. (**A**) Comparison of HS6ST2 expressions between tumors and normal samples in the TCGA database. (**B**) Comparison of HS6ST2 expressions between tumors and normal samples in the TCGA and the GTEx databases. (**C**) HS6ST2 expression in tumor cell lines. *P < 0.05, **P < 0.01, ***P < 0.001.

We initially conducted a pan-cancer analysis of 33 malignancies from the TCGA database to have a deeper knowledge of HS6ST2 is expressed in different cancer types. Significant variations in HS6ST2 expression were detected between para-cancerous and tumor tissues in 13 kinds of cancer, and malignancies without matched normal samples were excluded (Supplementary Table S1). HS6ST2 was highly expressed in bladder urothelial carcinoma (BLCA), cholangiocarcinoma (CHOL), pheochromocytoma and paraganglioma (PCPG), colon adenocarcinoma (COAD), esophageal carcinoma (ESCA), LUAD, thyroid carcinoma (THCA), and lung squamous cell carcinoma (LUSC). In contrast, tumor tissues had lower levels of HS6ST2 than healthy tissues in breast invasive carcinoma (BRCA), kidney renal papillary cell carcinoma (KIRP), uterine corpus endometrial carcinoma (UCEC), kidney renal clear cell carcinoma (KIRC), and kidney chromophobe (KICH) (Fig. 1A). Because the scarcity of normal samples for certain malignancies, we employed dataset from the GTEx database that contains data for a variety of healthy tissues from individuals. When we compared HS6ST2 expression levels from the GTEx and TCGA datasets, we identified significant differences in 19 of the 26 cancer types (Supplementary Table S1). HS6ST2 expression was lower in acute myeloid leukemia (LAML), adrenocortical carcinoma (ACC), ovarian serous cystadenocarcinoma (OV), pancreatic adenocarcinoma (PAAD), KICH, KIRC, KIRP, and stomach adenocarcinoma (STAD) but higher in the other 11 tumors (Fig. 1B). Furthermore, we utilized the CCLE database for investigating HS6ST2 expression of several cancer cell lines (Fig. 1C). Compared to para-carcinoma tissues, HS6ST2 expression was upregulated in numerous tumors, and thus, HS6ST2 may function as an oncogene in these malignancies.

### HS6ST2 is associated with DNA methylation and the expression of RNA methylation-associated genes across cancers

**Figure 2:**
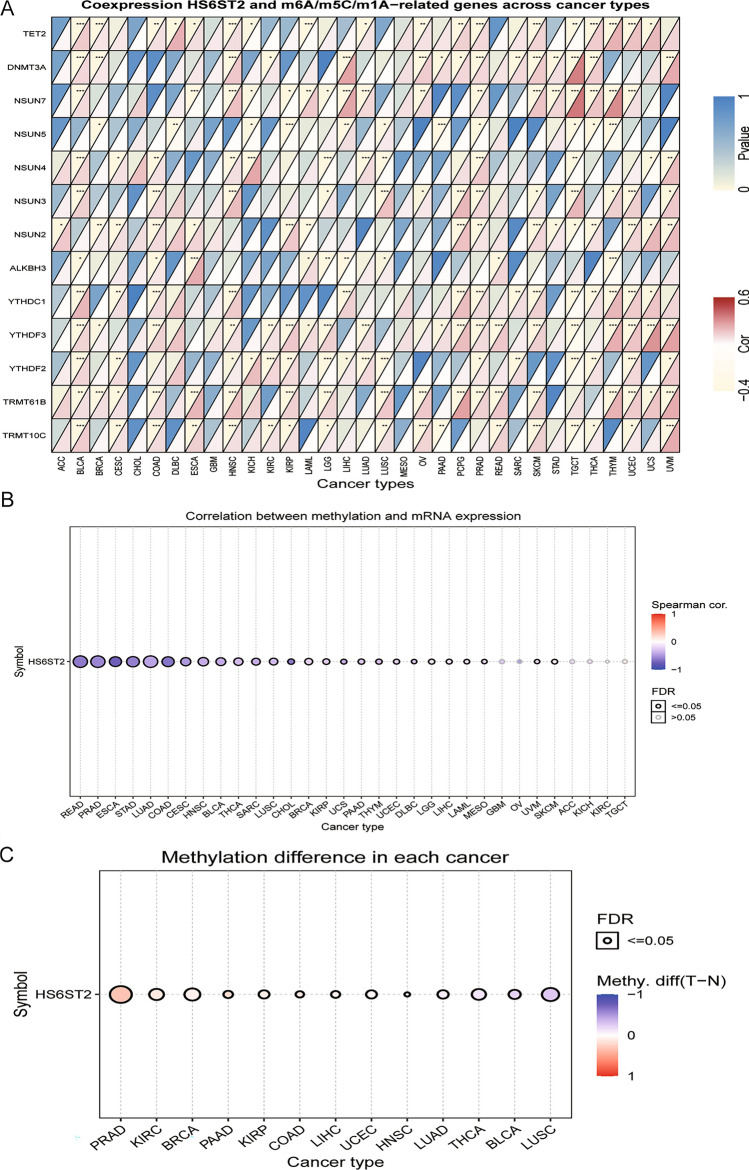
Associations between HS6ST2 expression and m6A, m5C, m1A-related genes, and DNA methylation in pan-cancer. (**A**) Heatmap illustrating the relationship between HS6ST2 and m6A, m5C, m1A-related genes. The top left triangle represents the P-value, and the bottom right triangle represents the correlation coefficient. *P < 0.05, **P < 0.01, ***P < 0.001. (**B**) Correlation between methylation and mRNA expression of HS6ST2. (**C**) Methylation difference of HS6ST2 mRNA in different cancers.

RNA methylation modification, including m1A, m5C, and m6A, occurs in a variety of RNAs, with varying effects on RNA function^29^. Positive associations were discovered between HS6ST2 and TET2 and DNMT3A in BLCA, prostate adenocarcinoma (PRAD), BRCA, head and neck squamous cell carcinoma (HNSC), THCA, and skin cutaneous melanoma (SKCM) when we calculated their expression levels with respect to other RNA methylation-related genes (Supplementary Table S1). HS6ST2 expression was negatively correlated with NSUN4 and YTHDF2 in LUSC, testicular germ cell tumors (TGCT), and brain lower grade glioma (LGG) but positively correlated with NSUN4 and YTHDF2 in BLCA, cervical squamous cell carcinoma and endocervical adenocarcinoma (CESC), HNSC, KICH, uveal melanoma (UVM), LUAD, and PRAD (Fig. 2A). Similarly, the expression of YTHDC1 and YTHDF3 was negatively correlated with HS6ST2 in TGCT, but positively correlated with HS6ST2 in BLCA, COAD, UCEC, HNSC, PCPG, PRAD, SKCM, uterine carcinosarcoma (UCS), and thymoma (THYM).

Changes in DNA methylation have been identified in a variety of malignancies and are considered a cause of carcinogenesis^30^. By evaluating the relationship between promoter methylation and HS6ST2 expression in GSCA data, significant relationships between gene expression and methylation were found in 26 distinct cancer types (Fig. 2B). Figure 2C shows that HS6ST2 expression was positively correlated with promoter methylation in BLCA, THCA, and LUAD, LUSC but negatively correlated with promoter methylation in BRCA, UCEC, HNSC, COAD, liver hepatocellular carcinoma (LIHC), PAAD, KIRC, KIRP, and PRAD (Supplementary Table S1). The findings indicate that an epigenetically altered state of HS6ST2 may facilitate carcinogenesis.

### Prognostic significance of HS6ST2

**Figure 3:**
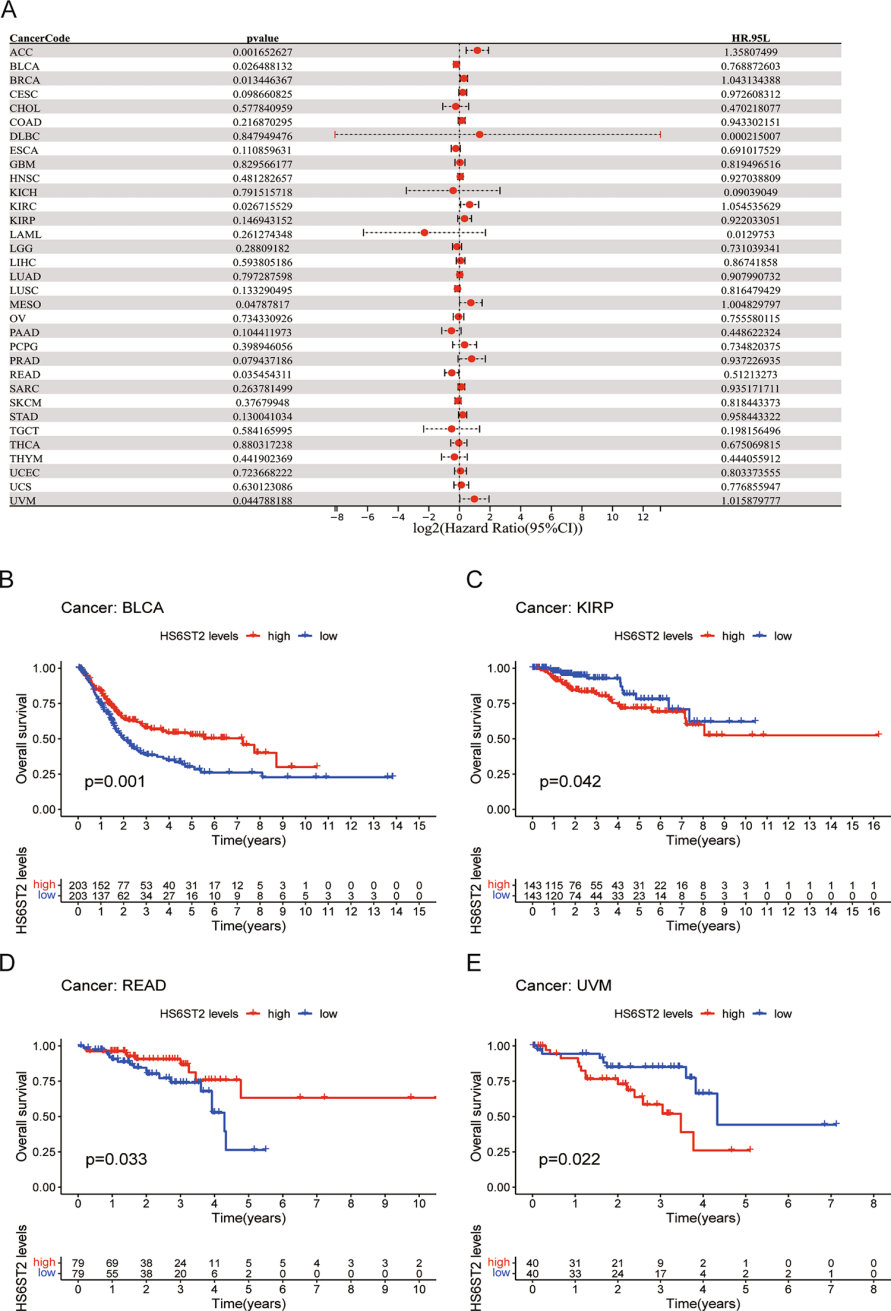
Association between HS6ST2 expression and overall survival (OS). (**A**) Forest plot shows the univariate cox regression results for the association between HS6ST2 expression and OS in 33 types of tumors. (**B**–**E**) Kaplan–Meier analysis of the association between HS6ST2 expression and OS.

**Figure 4:**
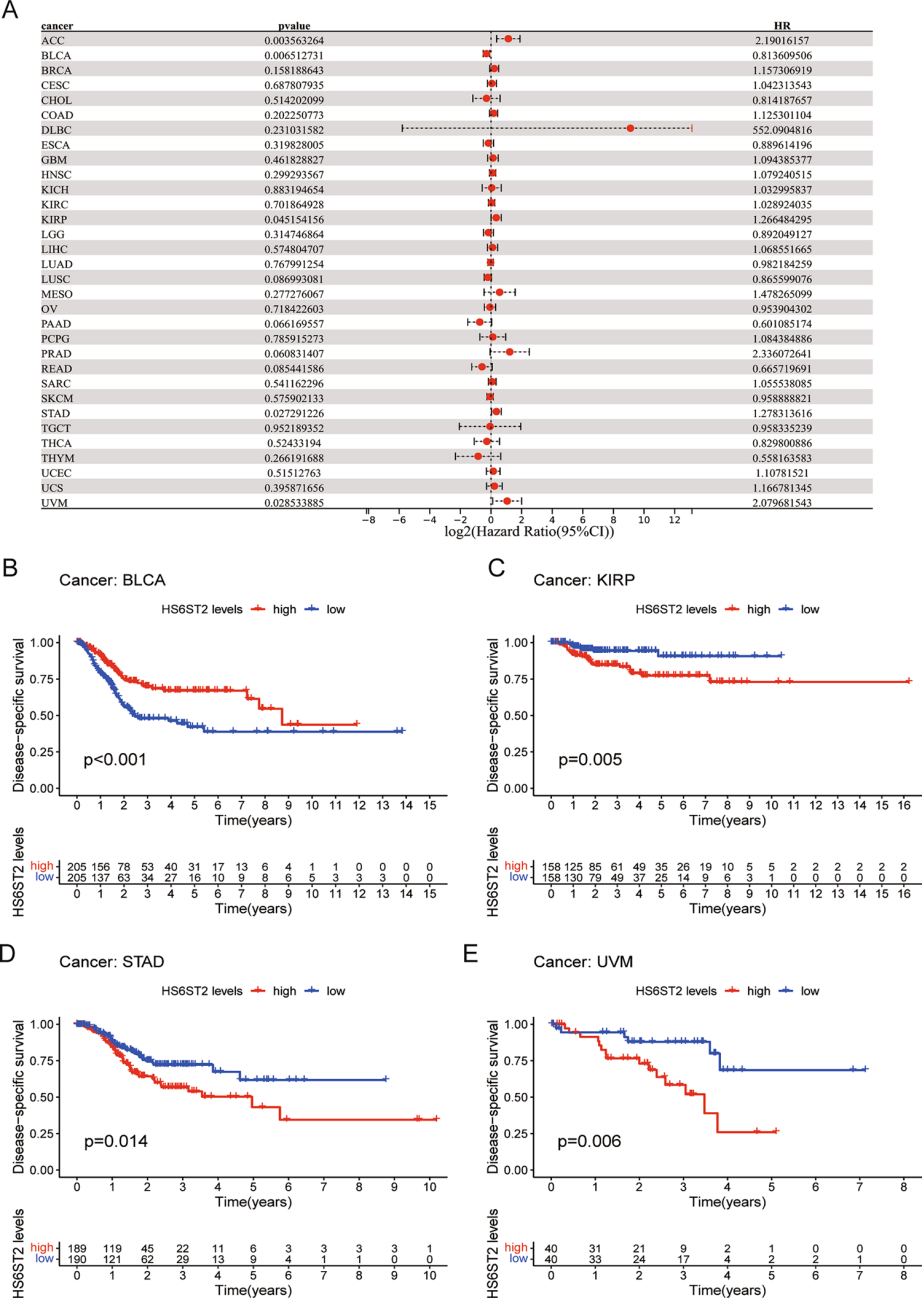
Association between HS6ST2 expression and disease-specific survival (DSS). (**A**) Forest plot shows the univariate cox regression results for the association between HS6ST2 expression and DSS ​in 33 types of tumors. (**B**–**E**) Kaplan–Meier analysis of the association between HS6ST2 expression and DSS.

Via the TCGA database, we examined the value of HS6ST2 mRNA expression for predicting OS, DFI, DSS, and PFI across cancers. Cox regression analysis revealed that low HS6ST2 expression was connected with poorer OS in breast invasive carcinoma, mesothelioma, kidney renal clear cell carcinoma, uveal melanoma, and adrenocortical carcinoma but associated with better OS in rectum adenocarcinoma and bladder urothelial carcinoma (Fig. 3A). As determined by KM OS analysis, HS6ST2 was a protective factor for patients with bladder urothelial carcinoma and rectum adenocarcinoma but a risk factor for patients with kidney renal papillary cell carcinoma and uveal melanoma (Fig. 3B–E). We also determined the correlation between the expression of HS6ST2 and the DFS rate in 33 different cancers. HS6ST2 was found to be a protective factor in bladder urothelial carcinoma and a risk factor in kidney renal papillary cell carcinoma, stomach adenocarcinoma, adrenocortical carcinoma, and according to Cox regression analyses (Fig. 4A). Similarly, KM analysis of DSS uncovered that HS6ST2 was a risk factor in stomach adenocarcinoma, kidney renal papillary cell carcinoma, and uveal melanoma but acted as a protective factor in bladder urothelial carcinoma (Fig. 4B–E).

**Figure 5:**
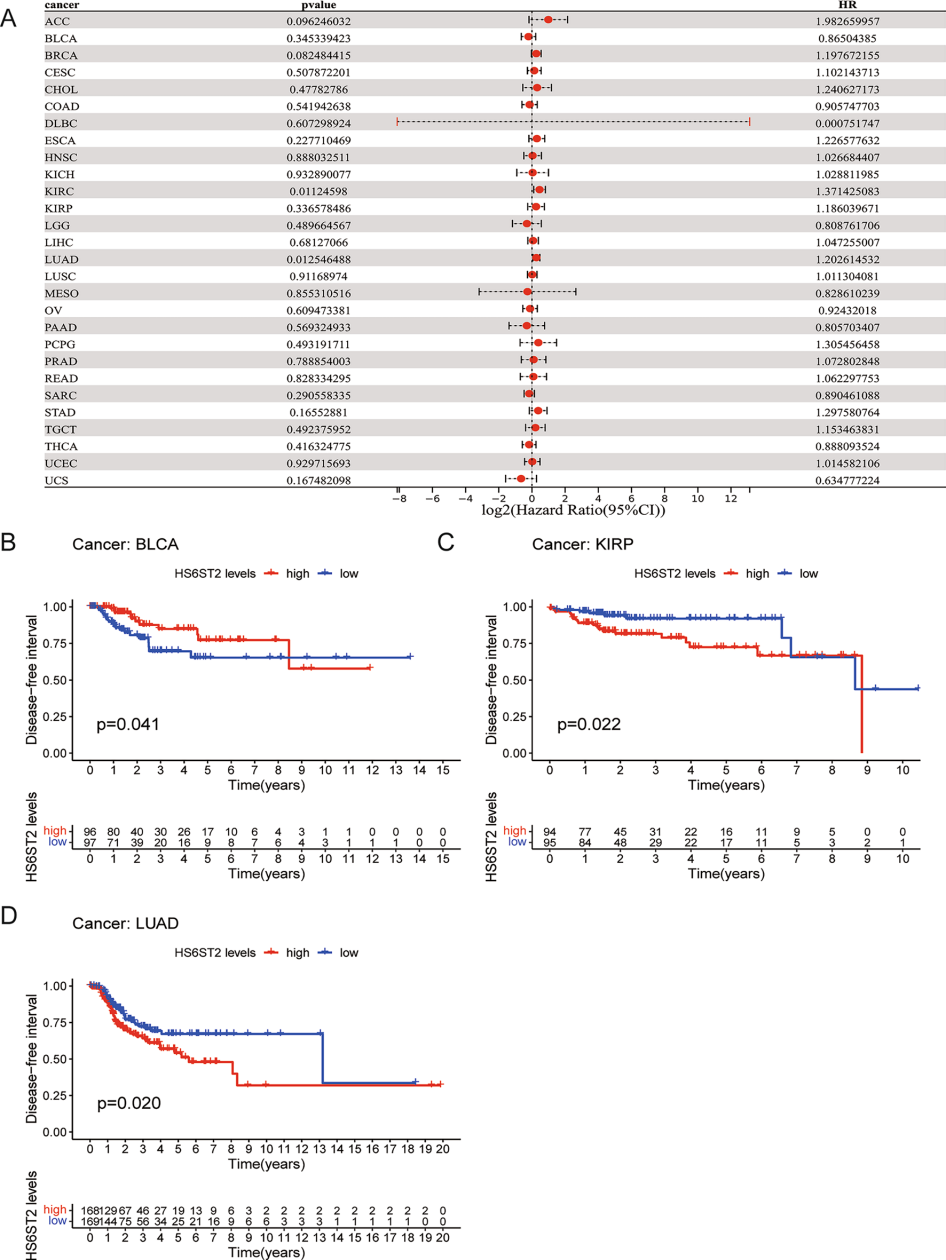
Association between HS6ST2 expression and disease-free interval (DFI). (**A**) Forest plot shows the univariate cox regression results for the association between HS6ST2 expression and DFI ​in 33 types of tumors. (**B**–**D**) Kaplan–Meier analysis of the association between HS6ST2 expression and DFI.

**Figure 6:**
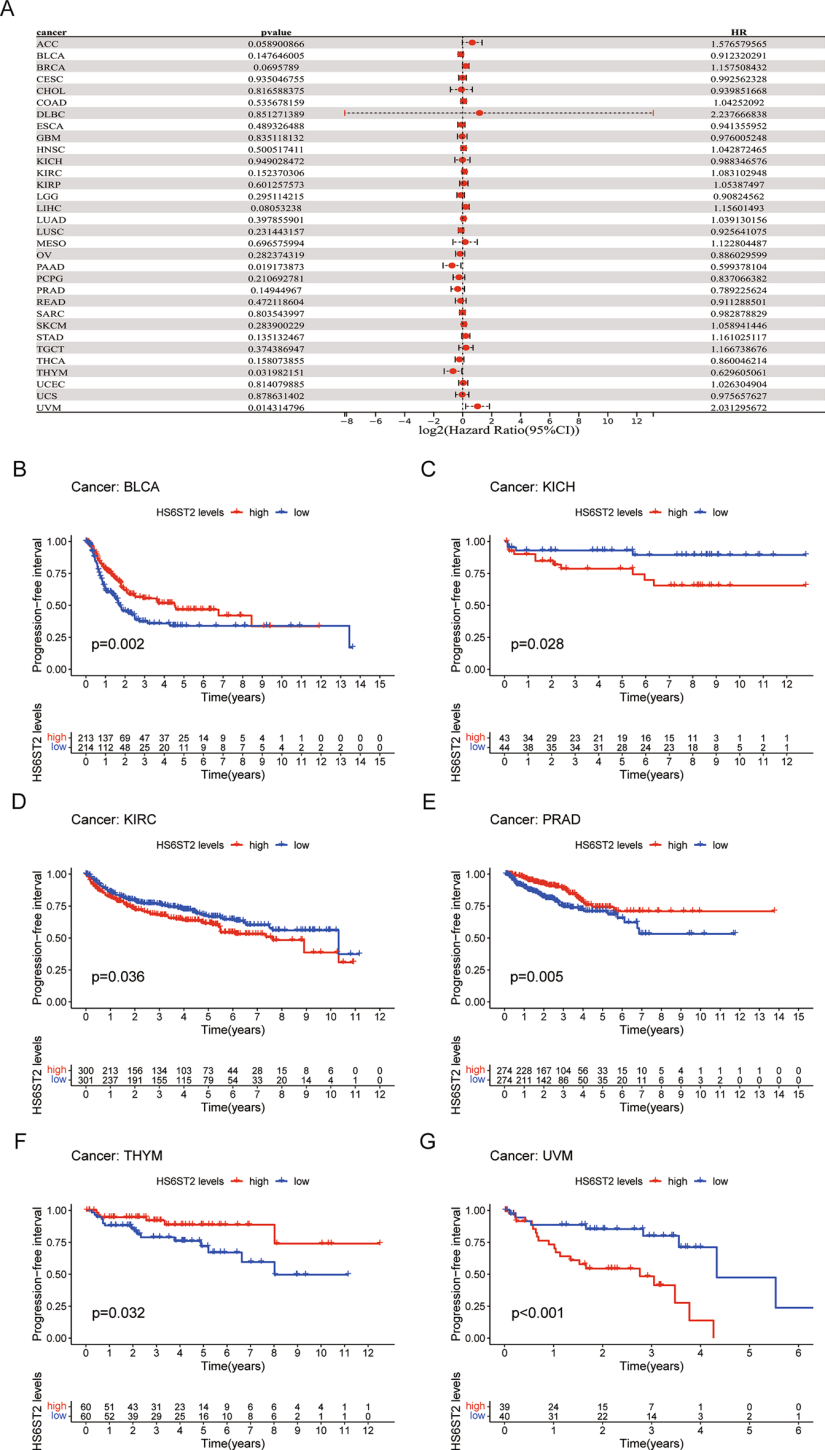
Association between HS6ST2 expression and progression-free interval (PFI). (**A**) Forest plot shows the univariate cox regression results for the association between HS6ST2 expression and PFI ​in 33 types of tumors. (**B**–**G**) Kaplan–Meier analysis of the association between HS6ST2 expression and PFI.

To evaluate the efficacy of radical surgery, the DFI was assessed, and this index was then used to predict the median time to recurrence. KM and Cox regression analyses showed that high HS6ST2 expression was associated with worse DFI in lung adenocarcinoma and kidney renal papillary cell carcinoma and with better DFI in bladder urothelial carcinoma (Fig. 5A–D). In addition, the correlation between PFI and HS6ST2 expression was examined to assess the responsiveness of cancers to palliative care. Cox regression analysis showed that HS6ST2 over-expression was associated with better PFI in pancreatic adenocarcinoma and thymoma but associated with poor PFI in uveal melanoma (Fig. 6A). According to the results of the KM analysis of PFI, HS6ST2 is a risk factor for patients with kidney renal clear cell carcinoma, kidney chromophobe, and uveal melanoma and a protective factor for bladder urothelial carcinoma, prostate adenocarcinoma, and thymoma (Fig. 6B–G). Our results demonstrated a significant correlation between HS6ST2 expression and outcomes in many types of cancer.

### Correlations of MMR gene expression, MSI status, and TMB with HS6ST2 expression levels

**Figure 7:**
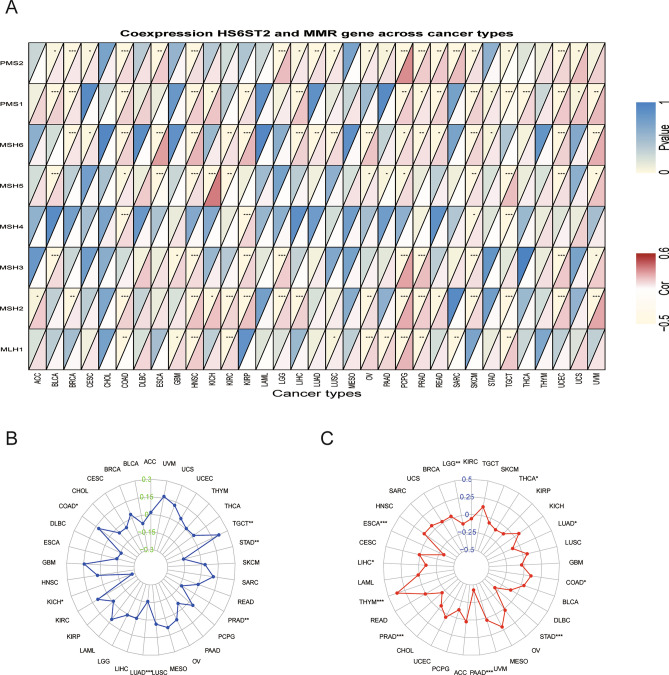
Associations between HS6ST2 expression and mismatch repair (MMR) gene, microsatellite instability (MSI), and tumor mutational burden (TMB) in pan-cancer. (**A**) Heatmap illustrating the relationship between HS6ST2 and MMR gene. The top left triangle represents the P-value, and the bottom right triangle represents the correlation coefficient. *P < 0.05, **P < 0.01, ***P < 0.001. (**B**) Correlation between HS6ST2 expression and MSI across cancers. (**C**) Correlation between HS6ST2 expression and TMB across cancers. The value of black represents the range, and the curves of blue and red represent the correlation coefficients.

DNA replication will produce errors; however, MMR genes can identify and correct such errors^31^. Due to abnormalities in the MMR mechanism, tumors with high MSI are more likely to accumulate many mutations in cancer-associated genes and develop a high TMB^32^. Consequently, we investigated the correlations between HS6ST2 expression and MMR genes. MMR gene expression was strongly favorably connected with HS6ST2 expression in almost all cancers (except cholangiocarcinoma and acute myeloid leukemia) (Fig. 7A, Supplementary Table S1). In six types of tumors (lung adenocarcinoma, prostate adenocarcinoma, stomach adenocarcinoma, testicular germ cell tumors, colon adenocarcinoma, and kidney chromophobe), HS6ST2 expression was related to MSI status, as presented in Fig. 7B. TMB was significantly linked with HS6ST2 expression in esophageal carcinoma, thymoma, prostate adenocarcinoma, pancreatic adenocarcinoma, stomach adenocarcinoma, brain lower grade glioma, liver hepatocellular carcinoma, colon adenocarcinoma, lung adenocarcinoma, and thyroid carcinoma, as shown in Fig. 7C. Our findings imply that HS6ST2 participates a role in mediating cancer via regulation of DNA damage.

### Relationships between HS6ST2 expression and TME factors

**Figure 8:**
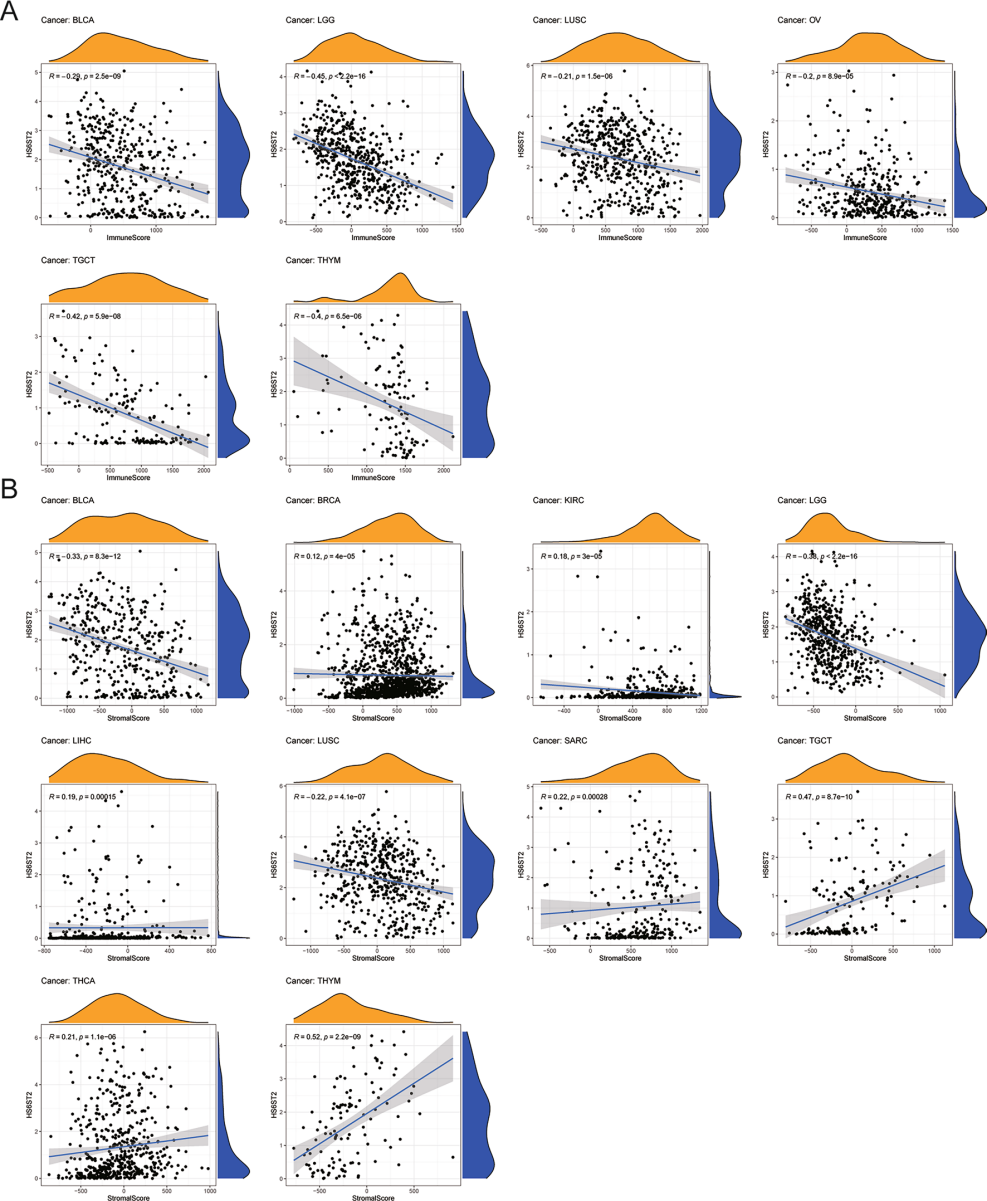
Correlation of HS6ST2 expression and the tumor microenvironment in various cancers. (**A**) Correlation between HS6ST2 and immune scores in BLCA, LGG, LUSC, OV, TGCT, and THYM. (**B**) Correlation between HS6ST2 and stromal scores in BLCA, BRCA, KIRC, LGG, LIHC, LUSC, SARC, TGCT, THCA, and THYM.

Accumulating evidence implies that the TME is necessary for tumor development and incidence^33^. Our pan-cancer study found a negative correlation between immune and stromal scores and HS6ST2 expression in lung squamous cell carcinoma, bladder urothelial carcinoma, and brain lower grade glioma (Supplementary Table S1). As seen in Fig. 8A,B, HS6ST2 expression was positively correlated with stromal scores in testicular germ cell tumors and thymoma and inversely correlated with immune scores, respectively. Our findings imply that HS6ST2 may affect the immunological tolerance of cancer by altering the TME.

### Association between the levels of tumor-infiltrating immune cells and HS6ST2 expression

**Figure 9:**
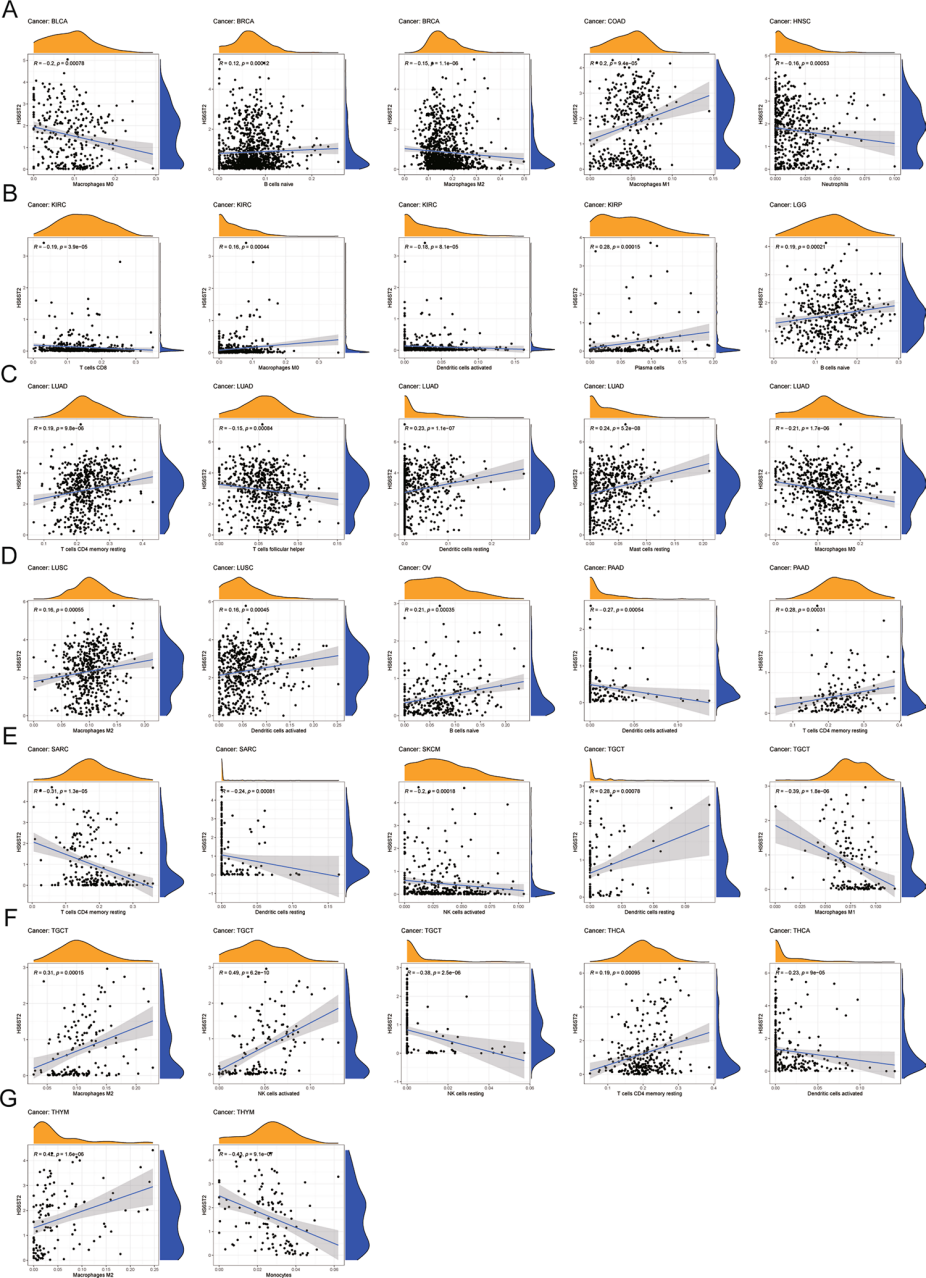
Correlation between HS6ST2 expression and tumor infiltration of different immune cells in the TCGA database. (**A**–**G**) Analysis of immune-associated cells infiltration with HS6ST2 expression in pan-cancer.

The association between HS6ST2 expression and the levels of 22 different tumor-infiltrating immune cells was then studied. HS6ST2 was found to be significantly linked with the infiltrating levels of naive B cells across cancers in the TCGA data, particularly in breast invasive carcinoma, brain lower grade glioma, and ovarian serous cystadenocarcinoma (Supplementary Table S1). In lung squamous cell carcinoma, testicular germ cell tumors, and thymoma, the number of M2 macrophages penetrating a tissue was strongly linked with HS6ST2 expression; however, in breast invasive carcinoma, the correlation was negative. We also examined the relationships between HS6ST2 and the levels of infiltrating T cells and natural killer (NK) cells in 33 cancer types. The level of infiltrating resting CD4 memory T cells was favorably correlated with HS6ST2 expression in lung adenocarcinoma, pancreatic adenocarcinoma, and thyroid carcinoma but inversely correlated with HS6ST2 expression in sarcoma. Furthermore, HS6ST2 was favorably connected with the level of activated NK cells activated in testicular germ cell tumors but inversely correlated with the level of activated NK cells in skin cutaneous melanoma. Figure 9 displays the correlations between HS6ST2 expression and the numbers of various types of invading immune cells in various cancers. Our results suggest that HS6ST2 may enhance immune system evasion by making it easier for immune cells to infiltrate tumors.

### The connection between HS6ST2 and immune-related gene expression

**Figure 10:**
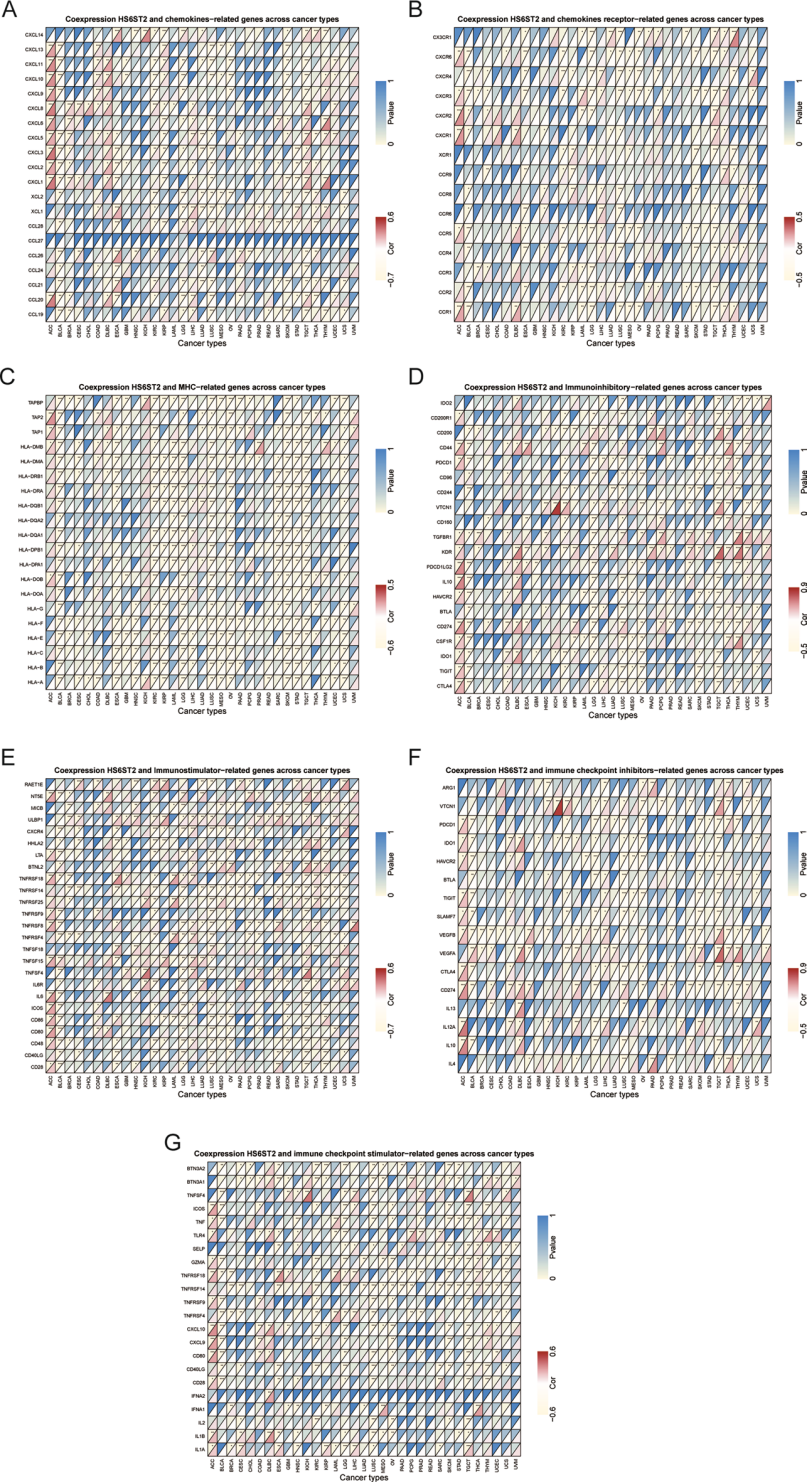
Co-expression of HS6ST2 and immune-related genes. *P < 0.05, **P < 0.01, ***P < 0.001.

Next, we used gene coexpression analysis to look for links between HS6ST2 expression and genes involved in the immune system in 33 different cancers. CXCL5, a gene linked to chemokines, was found to have a positive correlation with HS6ST2 expression in 16 tumor samples, showing a positive correlation in testicular germ cell tumors, breast invasive carcinoma, uterine corpus endometrial carcinoma, adrenocortical carcinoma, liver hepatocellular carcinoma, thymoma, colon adenocarcinoma, lymphoid neoplasm diffuse large b-cell lymphoma, and skin cutaneous melanoma but a negative correlation in bladder urothelial carcinoma, lung adenocarcinoma, kidney renal papillary cell carcinoma, thyroid carcinoma, lung squamous cell carcinoma, pancreatic adenocarcinoma, and esophageal carcinoma (Fig. 10A). As demonstrated in Fig. 10B,C, HS6ST2 expression was inversely correlated with CXCR3 and HLA-F in 13 malignancies (Supplementary Table S1). Additionally, we assessed the link between HS6ST2 and genes involved in immunological stimulation, immune suppression, and immune checkpoints. The findings showed a positive association between the expression of KDR and ULBP1 and HS6ST2 expression in 11 different types of cancer (in particular, kidney renal papillary cell carcinoma, sarcoma, skin cutaneous melanoma, thyroid carcinoma, and uterine corpus endometrial carcinoma) (Fig. 10D,E). On the contrary, the expression levels of VEGFB and TNFRSF14 were negatively correlated with HS6ST2 in breast invasive carcinoma, cervical squamous cell carcinoma and endocervical adenocarcinoma, lung squamous cell carcinoma, brain lower grade glioma, ovarian serous cystadenocarcinoma, skin cutaneous melanoma, sarcoma, thymoma, and uterine corpus endometrial carcinoma (Fig. 10F,G). Our findings indicate that HS6ST2 may have a role in tumor progression and immune evasion through the regulation of immune-related genes.

### The correlation between HS6ST2 expression and predicted treatment sensitivity

**Figure 11:**
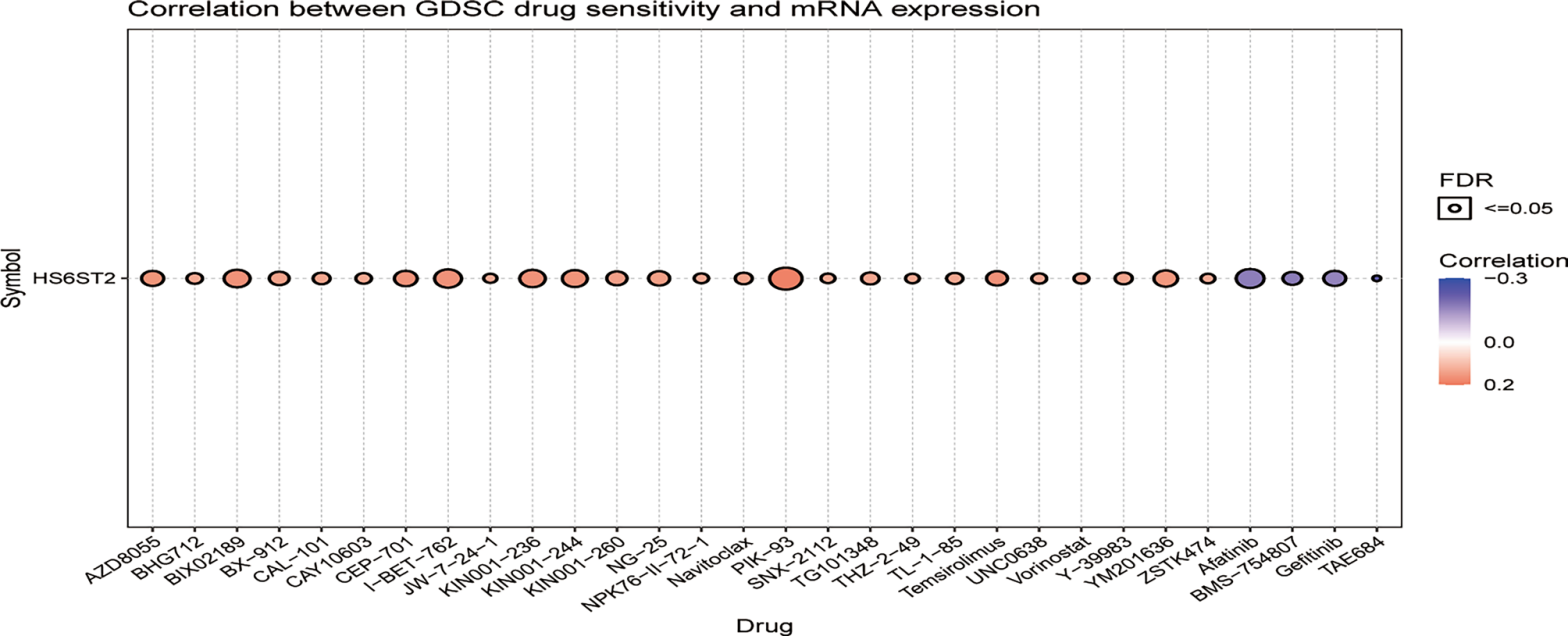
Relationship between HS6ST2 mRNA expression and drug sensitivity in the GDSC database. Figure summarizes the correlation between HS6ST2 expression and the top 30 sensitivity drugs in pan-cancer.

Analysis via the GDSC database disclosed a substantial association between HS6ST2 mRNA expression and predicted response to 78 anticancer treatments (Supplementary Table S1). The expression of HS6ST2 was positively linked with drug sensitivity in most malignancies, such as PIK-93, I-BET-762, BIX02189, KIN001-236, CEP-701, KIN001-244, AZD8055, YM201636, NG-25, and KIN001-260 (Fig. 11). In contrast, there was an inverse relationship between HS6ST2 expression and sensitivity to four small molecules and drugs: afatinib, BMS-754807, TAE684, and gefitinib. According to these results, HS6ST2 may be a possible therapeutic target for malignancies.

### Functional analysis of HS6ST2

**Figure 12:**
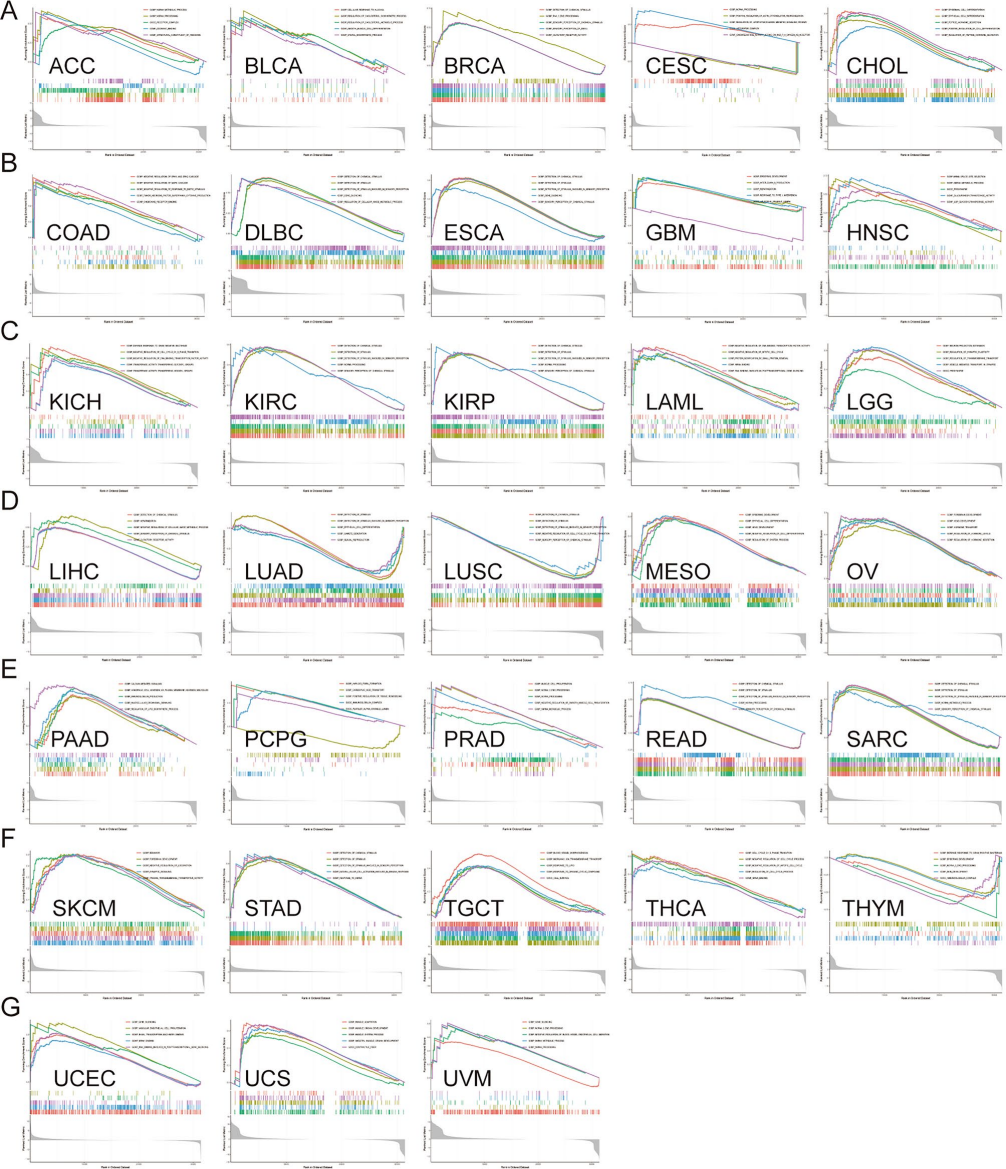
Results of GSEA. (**A**–**G**) GO functional annotation of HS6ST2 in various cancers. Curves of different colors show different functions or pathways regulated in different cancers. Peaks on the upward curve indicate positive regulation and peaks on the downward curve indicate negative regulation.

To ascertain the biological character of HS6ST2 in a range of tumor tissues, GSEA was performed. The resulting GO function is shown in Fig. 12. The data revealed a positive correlation of HS6ST2 with the detection of chemical stimuli in breast invasive carcinoma, lymphoid neoplasm diffuse large b-cell lymphoma, esophageal carcinoma, kidney renal clear cell carcinoma, liver hepatocellular carcinoma, sarcoma, stomach adenocarcinoma, kidney renal papillary cell carcinoma, and rectum adenocarcinoma, but the correlation was negative in lung squamous cell carcinoma. Similarly, GO BP analysis revealed a favorably correlation between HS6ST2 expression and ncRNA processing in adrenocortical carcinoma, kidney renal clear cell carcinoma, kidney renal papillary cell carcinoma, prostate adenocarcinoma, cervical squamous cell carcinoma and endocervical adenocarcinoma, and rectum adenocarcinoma. As seen in Fig. 12B,G, HS6ST2 correlated favorably with the degree to which genes were silenced in uterine corpus endometrial carcinoma, uveal melanoma, lymphoid neoplasm diffuse large b-cell lymphoma, and esophageal carcinoma. In contrast, HS6ST2 was negatively correlated with epithelial cell differentiation in lung adenocarcinoma, but the correlation was positive in cholangiocarcinoma and mesothelioma.

**Figure 13:**
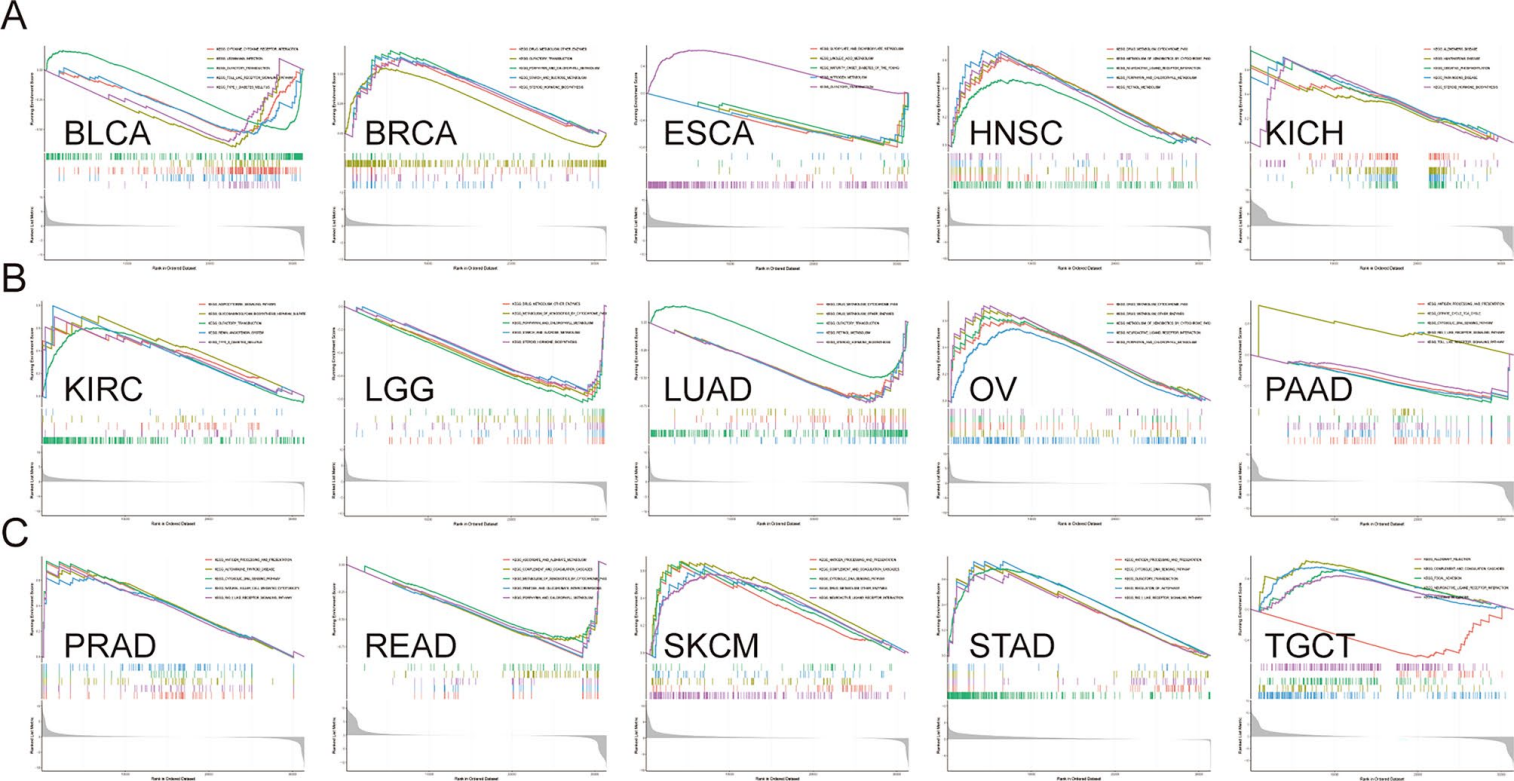
Results of GSEA. (**A**–**C**) KEGG pathway analysis of HS6ST2 in multiple cancers. Peaks on the upward curve indicate positive regulation and peaks on the downward curve indicate negative regulation.

The top five KEGG pathways significantly connected with HS6ST2 expression in each tumor are shown in Fig. 13. Positive correlations were found between HS6ST2 expression and the cytosolic DNA sensing pathway and antigen processing and presentation in prostate adenocarcinoma, skin cutaneous melanoma, and stomach adenocarcinoma, but the correlations were negative in pancreatic adenocarcinoma. In contrast, in rectum adenocarcinoma and brain lower grade glioma, HS6ST2 expression was inversely correlated with the porphyrin and chlorophyll metabolism pathways and the metabolism of xenobiotics by cytochrome P450, while the correlation was positive in head and neck squamous cell carcinoma. As shown in Fig. 13A,B, the Toll-like receptor signaling pathway was negatively correlated with HS6ST2 expression in bladder urothelial carcinoma and pancreatic adenocarcinoma. In addition, HS6ST2 was favorably connected with steroid hormone biosynthesis and drug metabolism in breast invasive carcinoma, while the correlation was negative in lung adenocarcinoma and brain lower grade glioma.

### HS6ST2 is upregulated in LUAD

**Figure 14:**
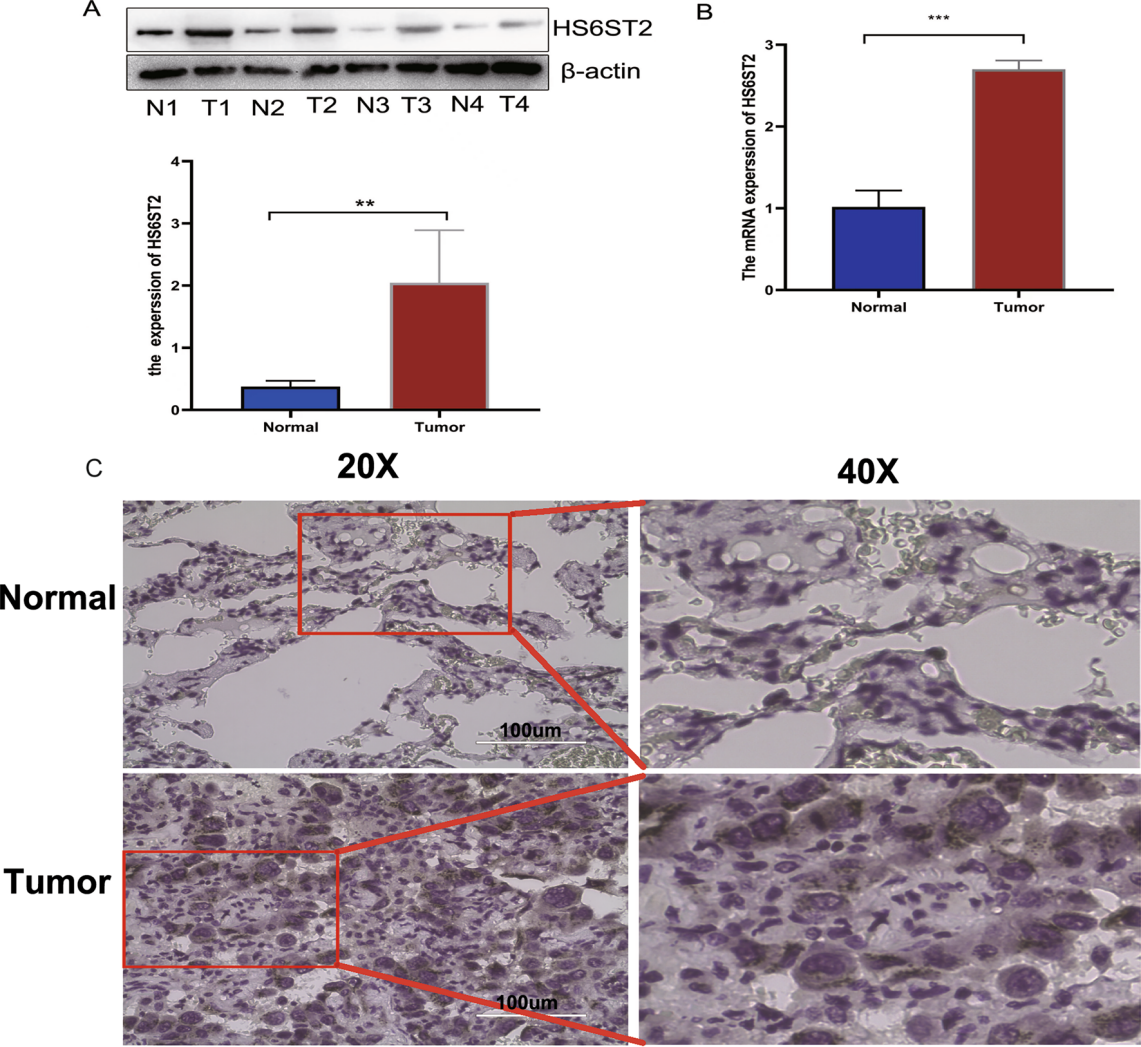
The expression of HS6ST2 in lung adenocarcinoma (LUAD). (**A**) Western blot analysis the expression of HS6ST2 level was evaluated in LUAD tissues. (**B**) The expression level of HS6ST2 mRNA was evaluated in LUAD tissues by qRT-PCR. (**C**) Representative images of different IHC staining intensities of HS6ST2 in LUAD tissues. Scale bars represent 100 μm.

To validate the aforementioned findings, we employed relevant molecular biology experimental methods. Firstly, we utilized the Western Blot technique to assess the expression levels of the HS6ST2 protein in lung adenocarcinoma tissue compared to the corresponding adjacent normal tissue. Grayscale analysis was conducted using ImageJ software. As depicted in Fig. 14A, the results indicated that the expression of HS6ST2 protein in lung adenocarcinoma tissue was significantly higher than in the corresponding adjacent normal tissue (**p < 0.01). Subsequently, we conducted RT-PCR experiments to examine the expression levels of HS6ST2 RNA in lung adenocarcinoma tissue compared to the corresponding adjacent normal tissue. As shown in Fig. 14B, the mRNA expression level of HS6ST2 in lung adenocarcinoma tissue was notably higher than in normal tissue (***p < 0.001). Furthermore, we employed immunohistochemistry experiments to analyze the expression level of HS6ST2 in lung adenocarcinoma samples (Fig. 14C). Immunostaining results revealed that HS6ST2 protein was primarily expressed in the cytoplasm of cells. The staining intensity was weaker in normal tissue compared to adenocarcinoma tissue, and the staining area was larger in lung adenocarcinoma tissue. These results collectively suggest that the expression level of HS6ST2 in lung adenocarcinoma tissue is higher than that in adjacent normal tissue.

## Discussion

Pan-cancer studies provide a comprehensive understanding of the functional characteristics and molecular aberrations of many cancer types and are useful for identifying new biomarkers for early cancer detection and therapeutic targets^34^. HS sulfotransferases such as HS6ST2 catalyze the transfer of sulfate to HS^7^. Accumulating data suggest that HS6ST2 is related to the formation and progression of numerous types of tumors^11–14^. Nevertheless, HS6ST2 has not been researched thoroughly in the context of cancer, and its role in carcinogenesis and across cancers remains uncertain. On the basis of data from various databases, a pan-cancer assessment of HS6ST2 expression in 33 distinct malignancies was conducted, and its expression in LUAD tissues was validated.

The significance of HS6ST2 expression and prognosis relevance in different malignancies were first examined. In line with other prior investigations, ours indicated that HS6ST2 was dysregulated in 13 distinct cancer types^14,15,20,22,23^ but inconsistent with one study^18^. For instance, HS6ST2 is overexpressed in thyroid cancer, and HS6ST2 overexpression is correlated with the progression of thyroid cancer^14,15^. Silencing of HS6ST2 reduced the transcriptional activity mediated by Smad2/3/4, thereby inhibiting the production of IL-11 induced by TGF-β, resulting in the inhibition of breast tumor growth^20^. Another study illustrated that HS6ST2 was upregulated in NSCLC^22^ and promoted cell development in NSCLC^23^. Our findings in renal clear cell cancer contradict those of earlier studies^17^, which showed that HS6ST2 is a potential prognostic biomarker and that inhibition of HS6ST2 causes decreased migration and invasion of RCC cells^18^. This inconsistency is likely attributable to the fact that earlier studies placed a greater emphasis on RCC cell lines with high metastatic ability. In particular, HS6ST2 overexpression was related with better prognosis in THYM, BLCA, and READ, while HS6ST2 overexpression was associated with worse outcome in UVM, KIRP, and STAD. In line with this, HS6ST2 upregulation was found in gastric cancer and was inversely correlated with OS and favorably correlated with depth of distant metastasis, tumor invasion, and tumor-node-metastasis stage^35^. Our data imply that HS6ST2 can be utilized as a predictive biomarker for a range of cancers. The process of carcinogenesis is heavily influenced by alterations in DNA methylation and RNA methylation^29,30^. Our study found significant associations between DNA methylation and HS6ST2 expression within 13 distinct cancer types, including negative correlations in BRCA, COAD, HNSC, KIRP, KIRC, PAAD, PRAD, LIHC, and UCEC and positive correlations in BLCA, LUSC, LUAD, and THCA. In addition, HS6ST2 expression was favorably connected with DNMT3A, YTHDF2, NSUN4, and TET2, all of which are involved in the maintenance of m6A, m5C, and m1A modifications. High levels of MSI, caused by tumors with abnormalities in the MMR system, exacerbate TMB and ultimately lead to tumor development^32^. In our study, HS6ST2 expression was found to be favorably connected with TMB, and MSI, and MMR gene expression in most tumors. Consistent with previous studies, these results suggest that HS6ST2 mediates carcinogenesis by affecting MMR genes, TMB, and MSI status, all of which are related to DNA methylation and RNA methylation^29,30,32^.

According to our findings, HS6ST2 also plays a crucial role in cancer immunity. The TME is important for cancer progression and therapeutic response^33^. HS6ST2 expression was found to be inversely related to stromal and immune cell composition in the TME in LUSC, BLCA, and LGG, but not in THCA, as measured by ESTIMATE scores. Immune cells that invade tumors play an important role in maintaining stability within the TME and in the development and treatment of cancer^36,37^. The TME contains a large number of M2 macrophages, and naive B cells may provide support for tumor growth^38,39^. HS6ST2 had a positive relationship with the levels of M2 macrophages cells and naive B cells in many malignancies. Those findings may explain the function of HS6ST2 as a risk factor in the majority of tumor types. In addition, our research confirmed the coexpression of HS6ST2 with genes related to immunological function. The HS6ST2 expression level was significantly connected with immune-related genes involved in tumorigenesis but was negatively correlated with CXCR3 and HLA-F^40,41^. All of these findings suggest that modulating HS6ST2 expression may also be a viable method for improving immunotherapy outcomes.

Despite the emergence of newer technologies and tailored therapeutics, resistance to drugs remains a major problem for researchers in the laboratory and clinic, and new drug resistance-combating strategies have been developed, such as restoring the function of tumor suppressor genes^42^ and RNA interference^43^. We determined the correlation of HS6ST2 with the IC50 values of over 750 anti-cancer medications. The data showed that increased HS6ST2 expression was correlated with reduced sensitivity to numerous drugs, indicating its probable participation in medication resistance; in contrast, increase HS6ST2 expression was correlated with increased sensitivity to afatinib, BMS754807, gefitinib, and TAE684. This discovery suggests that modulating the expression of HS6ST2 may be a strategy for enhancing anticancer drug efficacy. Finally, our enrichment studies implied that HS6ST2 may influence the pathophysiology and/or etiology of cancer by functioning in RNA processing, gene silencing, epithelial cell differentiation, the cytosolic DNA sensing pathway, antigen processing and presentation, and/or drug metabolism. According to these results, HS6ST2 may regulate tumor development via these mechanisms. In addition, a number of experiments were conducted to evaluate the expression of HS6ST2 in LUAD tissues. HS6ST2 was increased in LUAD tissues compared to nearby normal tissues.

Our study has several limitations. Initially, our research revealed some inconsistent results regarding specific tumors. Thus, the expression and function of HS6ST2 must be investigated in a larger sample size. Second, according to our findings, HS6ST2 has the potential to be used as a prognostic factor in a wide range of malignancies; however, this hypothesis requires further investigation. Third, additional experimental and clinical validation of the effects of HS6ST2 on the TME and immunotherapy response is needed. Fourth, even though we determined the expression of HS6ST2 in LUAD tissues, the precise regulatory mechanisms remain obscure.

In summary, HS6ST2 is discrepancy expressed between malignant and para-cancerous tissues, and there are associations between HS6ST2 and RNA and DNA methylation, as revealed by our initial pan-cancer investigations. The results imply that HS6ST2 can be utilized as a potential indicator for a variety of cancer types. Furthermore, HS6ST2 expression was found to be correlated with immune cell infiltration, MSI status, and TMB across malignancy types, and the effect of HS6ST2 on tumor immunity differed between cancer types. These results will help researchers clarify the function of HS6ST2 in carcinogenesis and progression and serve as a basis for the development of improved specific and individualized immunotherapy.

### Supplementary Information

Supplementary Information 1.

Supplementary Information 2.

Supplementary Information 3.

Supplementary Information 4.

Supplementary Information 5.

Supplementary Information 6.

Supplementary Information 7.

Supplementary Legends.

Supplementary Tables.

Supplementary Information 8.

## Data Availability

The datasets generated and/or analysed during the current study are available in the [GTEx UCSC CCLE] repository, We obtained RNA expression dataset from health tissues in GTEx in addition to data on gene expression and somatic mutations for 33 malignancies from the UCSC Xena database (https://xenabrowser.net/datapages/). From the CCLE database, we obtained cell line RNA expression matrices (https://portals.broadinstitute.org/ccle/).

## Abbreviations

PD-1: Programmed death1
PD-L1: Programmed Death-Ligand 1
CTLA4: Cytotoxic T-Lymphocyte Associated Antigen 4
HLA: Human leukocyte antigen
β2MG: Beta-2 microglobulin
T cell: Thymus dependent lymphocyte
HS6ST2: Heparan sulfate 6-O-sulfotransferase 2
HSPGs: Heparan sulfate proteoglycans
HS: Heparan sulfate
SARS-CoV-2: Severe Acute Respiratory Syndrome Coronavirus 2
MMP13: Matrix Metalloproteinase 13
MAPK: Mitogen-activated proteinkinase
miR: MicroRNA
IL-8: Interleukin 8
FGF2: Fibroblast growth factor
OS: Overall survival
RCC: Renal cell carcinoma
Wnt: Wingless
TGF-β: Transforming growth factor-β
IL-11: Interleukin 11
EMT: Epithelial–mesenchymal transition
NSCLC: Non-small cell lung cancer
GTEx: Genotype-Tissue Expression
CCLE: Cancer Cell Line Encyclopedia
TCGA: The Cancer Genome Atlas
DNA: Deoxyribonucleic acid
ICI: Immune cell infiltration
MSI: Microsatellite instability
TMB: Tumor mutation burden
RNA: Ribonucleic Acid
MMR: Mismatch repair
GSEA: Gene set enrichment analysis
LUAD: Lung adenocarcinoma
GSCA: Gene Set Cancer Analysis
mRNA: Messenger RNA
m6A: N6-methyladenosine
m5C: Methyl-5-cytosine
m1A: N1-methyladenosine
KM: Kaplan‒Meier
DFI: Disease-free interval
DSS: Disease-specific survival
PFI: Progression-free interval
TME: Tumor microenvironment
CIBERSORT: Cell-type Identification By Estimating Relative Subsets Of RNA Transcripts
KEGG: Kyoto Encyclopedia of Genes and Genomes
GO: Gene Ontology
RT‒qPCR: Reverse Transcription-Quantitative Polymerase Chain Reaction
cDNA: Complementary DNA
IHC: Immunohistochemistry
DAB: 3,3'-Diaminobenzidine
HRP: Horseradish Peroxidase
ACC: Adrenocortical carcinoma
BLCA: Bladder Urothelial Carcinoma
BRCA: Breast invasive carcinoma
CESC: Cervical squamous cell carcinoma and endocervical adenocarcinoma
CHOL: Cholangiocarcinoma
COAD: Colon adenocarcinoma
DLBC: Lymphoid Neoplasm Diffuse Large B-cell Lymphoma
ESCA: Esophageal carcinoma
GBM: Glioblastoma multiforme
HNSC: Head and Neck squamous cell carcinoma
KICH: Kidney Chromophobe
KIRC: Kidney renal clear cell carcinoma
KIRP: Kidney renal papillary cell carcinoma
LAML: Acute Myeloid Leukemia
LGG: Brain Lower Grade Glioma
LIHC: Liver hepatocellular carcinoma
LUAD: Lung adenocarcinoma
LUSC: Lung squamous cell carcinoma
MESO: Mesothelioma
OV: Ovarian serous cystadenocarcinoma
PAAD: Pancreatic adenocarcinoma
PCPG: Pheochromocytoma and Paraganglioma
PRAD: Prostate adenocarcinoma
READ: Rectum adenocarcinoma
SARC: Sarcoma
SKCM: Skin Cutaneous Melanoma
STAD: Stomach adenocarcinoma
TGCT: Testicular Germ Cell Tumors
THCA: Thyroid carcinoma
THYM: Thymoma
UCEC: Uterine Corpus Endometrial Carcinoma
UCS: Uterine Carcinosarcoma
UVM: Uveal Melanoma
CBLL1: Cbl proto-oncogene like 1
WTAP: WT1 associated protein
METTL3: Methyltransferase 3 N6-adenosine-methyltransferase complex catalytic subunit
METTL14: Methyltransferase 14 N6-adenosine-methyltransferase complex catalytic subunit
RBM15: RNA binding motif protein 15
RBM15B: RNA binding motif protein 15 beta
ZC3H13: Zinc finger CCCH-type containing 13
ALKBH5: AlkB homolog 5, RNA demethylase
FTO: FTO alpha-ketoglutarate dependent dioxygenase
IGF2BP1: Insulin like growth factor 2 mRNA binding protein 1
YTHDC1: YTH domain containing 1
YTHDC12: YTH domain containing 2
YTHDF1: YTH N6-methyladenosine RNA binding protein 1
THDF2: YTH N6-methyladenosine RNA binding protein 2
YTHDF3: YTH N6-methyladenosine RNA binding protein 3
NSUN7: NOP2/Sun RNA methyltransferase family member 7
NSUN2: NOP2/Sun RNA methyltransferase 2
NSUN3: NOP2/Sun RNA methyltransferase 3
NSUN4: NOP2/Sun RNA methyltransferase 4
NSUN5: NOP2/Sun RNA methyltransferase 5
NSUN6: NOP2/Sun RNA methyltransferase 6
TRDMT1: TRNA aspartic acid methyltransferase 1
DNMT1: DNA methyltransferase 1
DNMT3: DNA methyltransferase 3
DNMT3A: DNA methyltransferase 3 alpha
DNMT3B: DNA methyltransferase 3 beta
NOP2: NOP2 nucleolar protein
TET2: Tet methylcytosine dioxygenase 2
TRMT6: TRNA methyltransferase 6 non-catalytic subunit
ALYREF: Aly/REF export factor
TRMT61A: TRNA methyltransferase 61A
TRMT61B: TRNA methyltransferase 61B
ALKBH1: AlkB homolog 1, histone H2A dioxygenase
ALKBH3: AlkB homolog 3, alpha-ketoglutarate dependent dioxygenase
M2: Membranous 2
NK: Natural killer
CD4: Cluster of differentiation 4
CXCL5: C-X-C motif chemokine ligand
CXCR3: C-X-C motif chemokine receptor 3
HLA-F: Major histocompatibility complex, class I, F
KDR: Kinase insert domain receptor
ULBP1: UL16 binding protein 1
VEGFB: Vascular endothelial growth factor B
TNFRSF14: TNF receptor superfamily member 14
GDSC: Genomics of Drug Sensitivity in Cancer
GO BP: GeneOntology Biological process
ncRNA: Non-coding RNA
Smad2/3/4: SMAD family member 2/3/4
B cell: B Lymphocyte
IC_50_: Half maximal inhibitory concentration
log2: Logarithm2
