# Supplementary figures and images for: Pancancer analysis of the correlations of HS6ST2 with prognosis, tumor immunity, and drug resistance
Source: Sci Rep. 2023 Nov 6;13:19209. doi: 10.1038/s41598-023-46525-x (PMC10628205; doi:10.1038/s41598-023-46525-x)

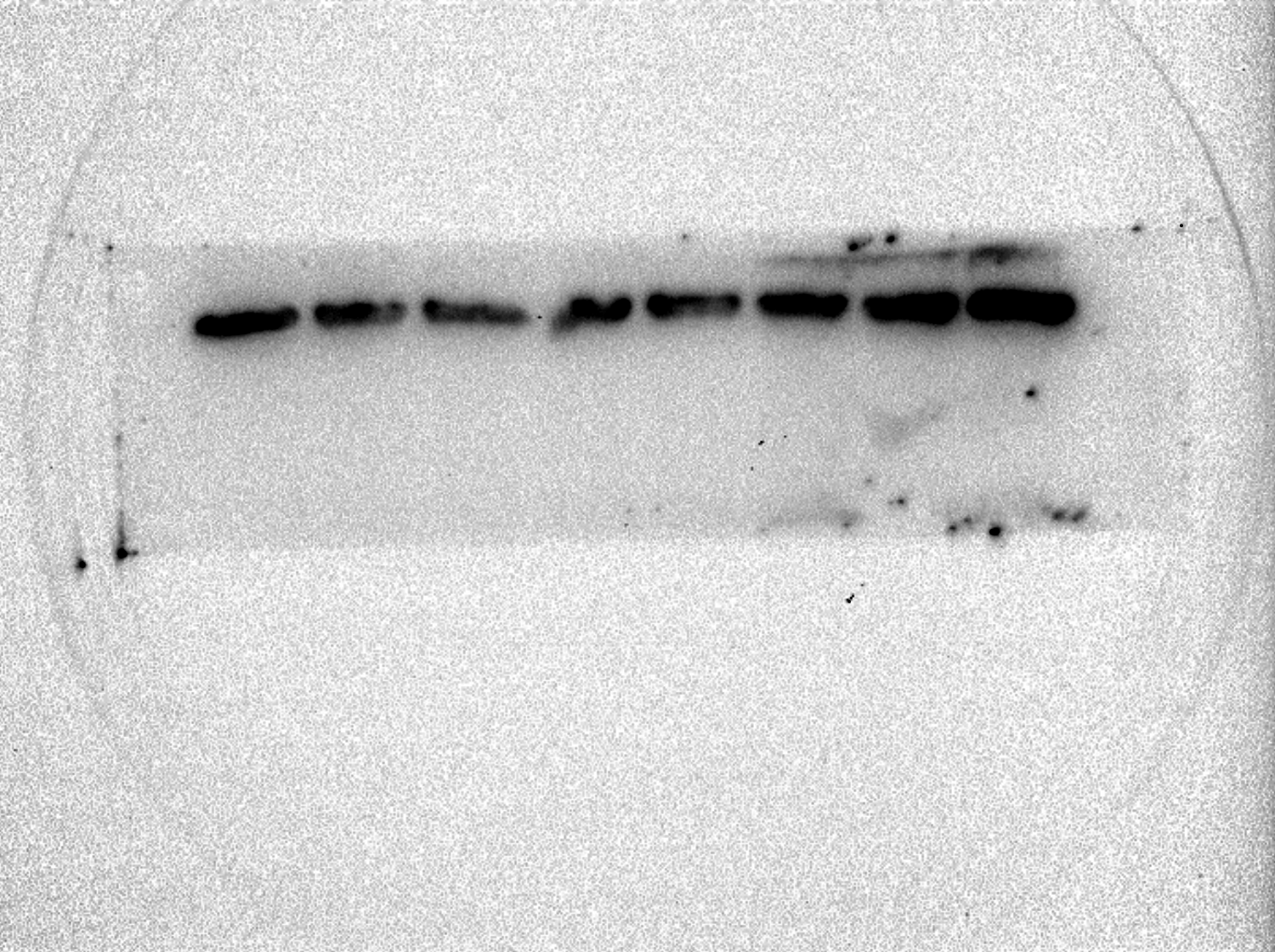

Supplement: Supplementary file 2 — Supplementary Information 2. [file 41598_2023_46525_MOESM2_ESM.tif]

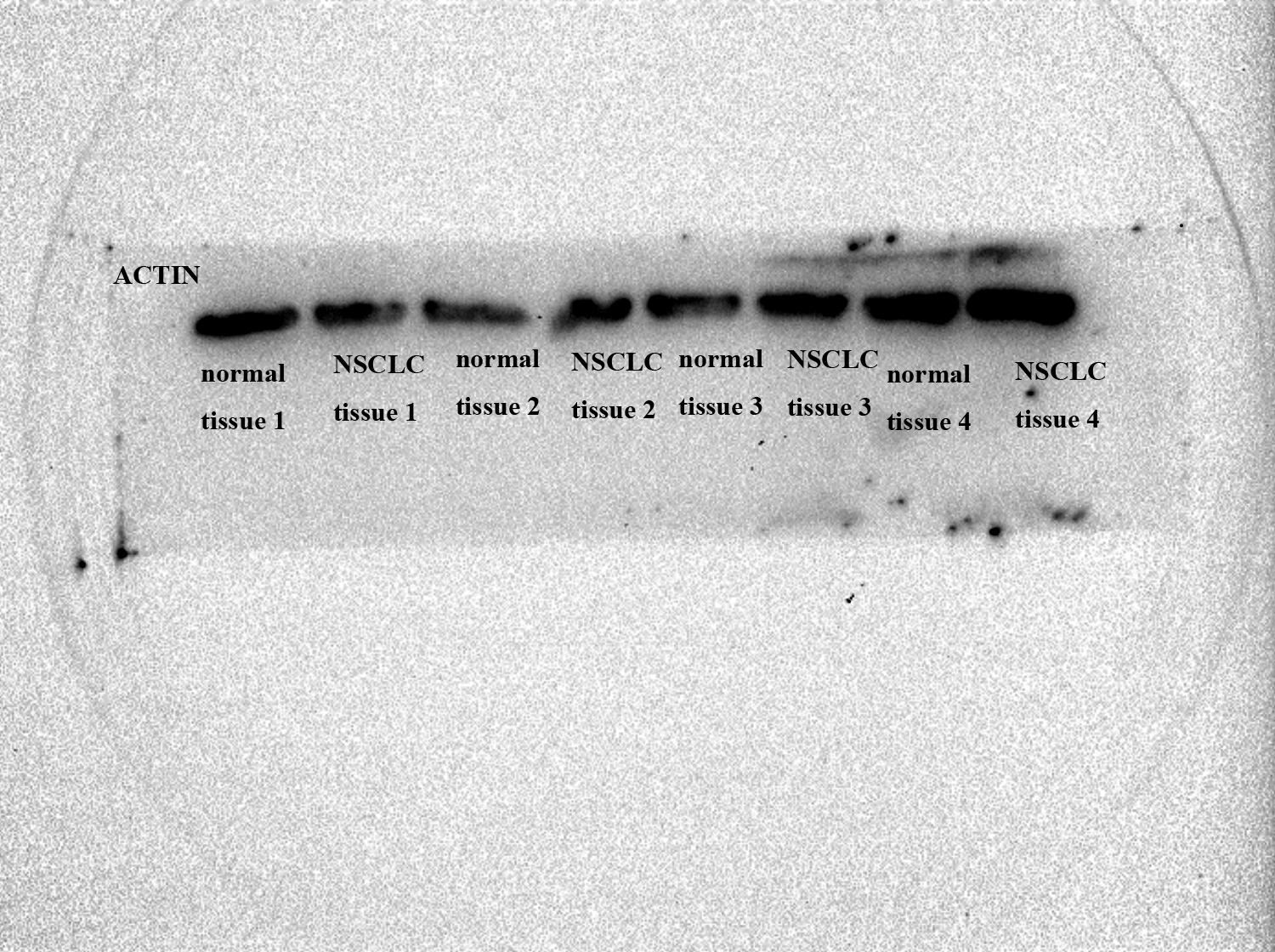

Supplement: Supplementary file 4 — Supplementary Information 4. [file 41598_2023_46525_MOESM4_ESM.tif]

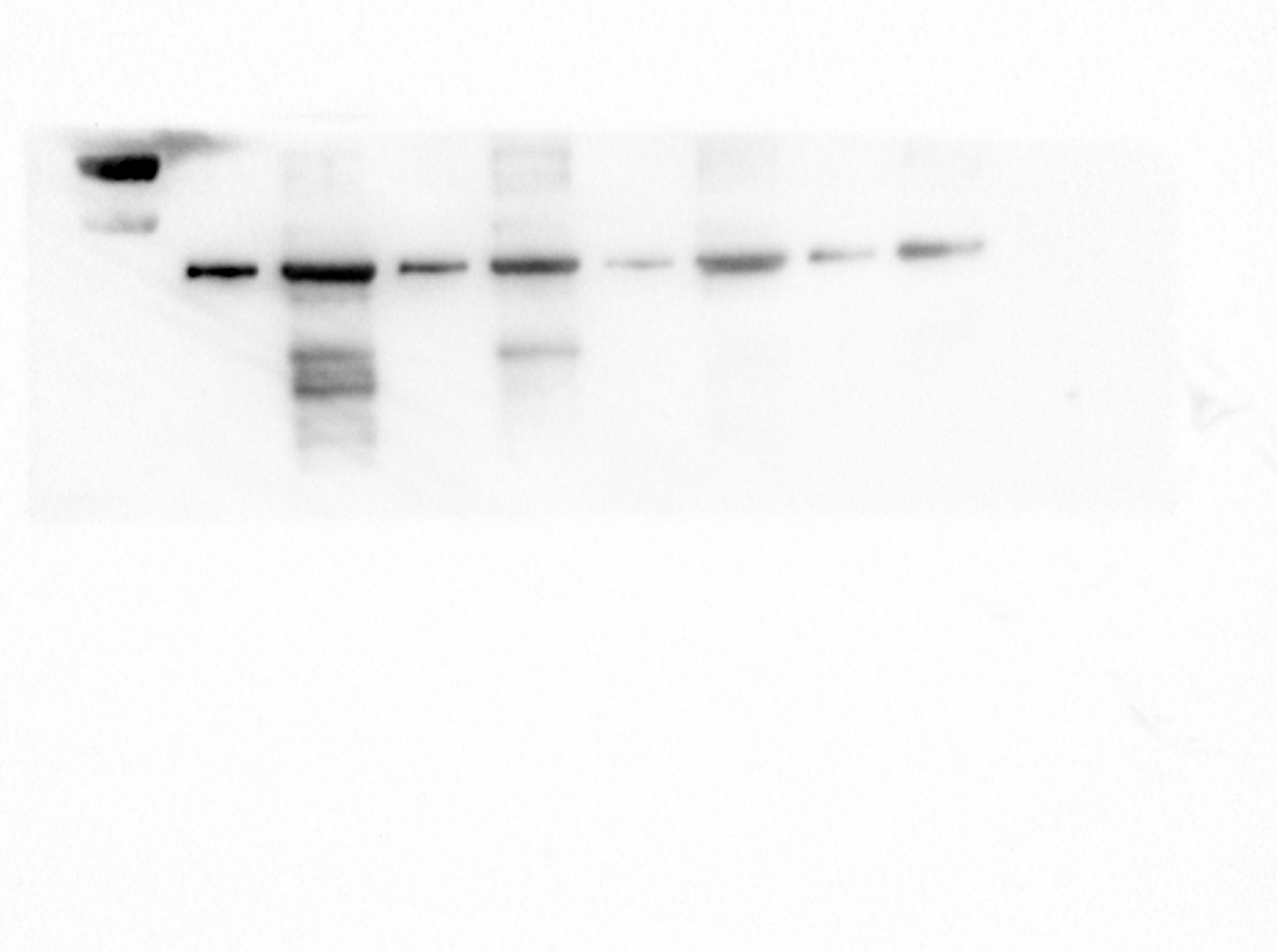

Supplement: Supplementary file 5 — Supplementary Information 5. [file 41598_2023_46525_MOESM5_ESM.tif]

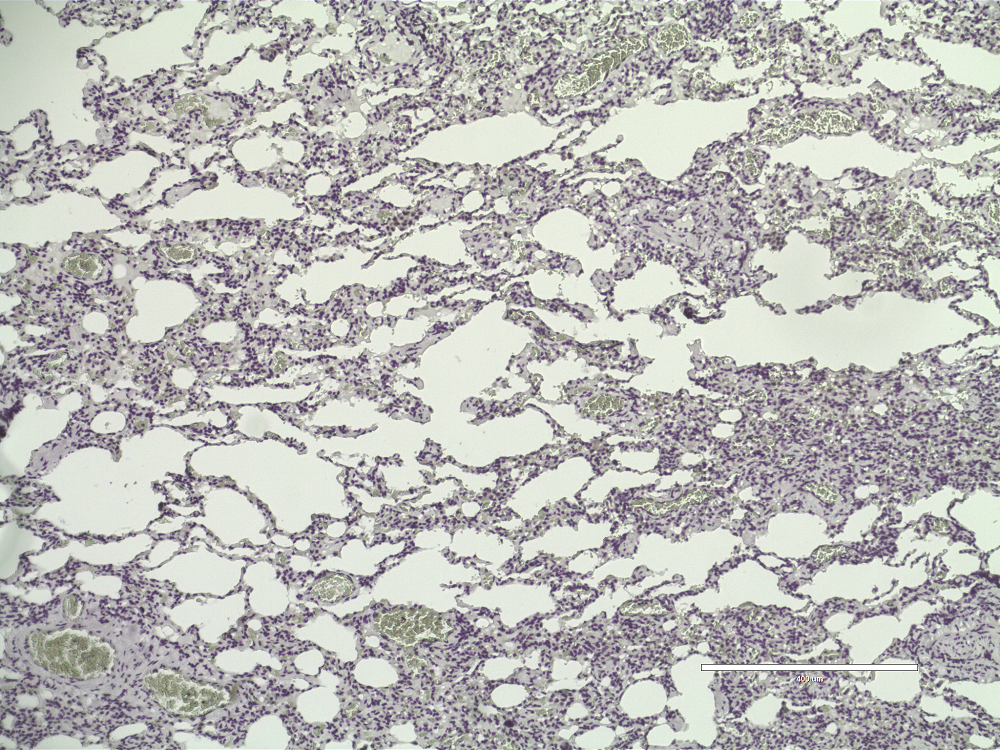

Supplement: Supplementary file 6 — Supplementary Information 6. [file 41598_2023_46525_MOESM6_ESM.tif]

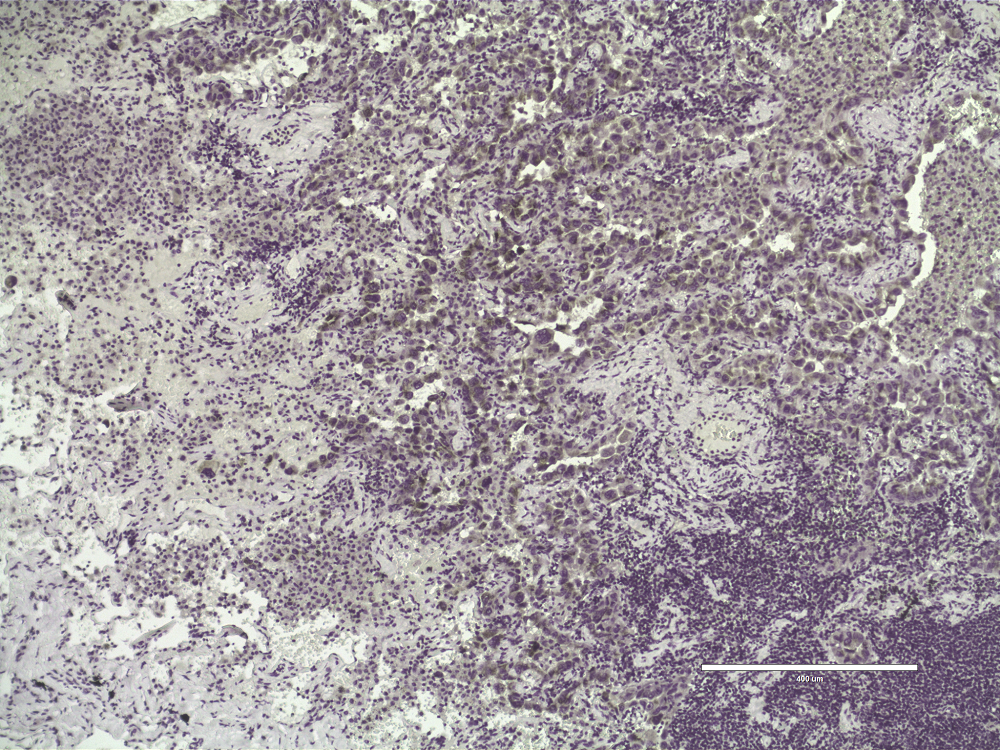

Supplement: Supplementary file 7 — Supplementary Information 7. [file 41598_2023_46525_MOESM7_ESM.tif]
