# Supplementary material for: Pancancer analysis of the correlations of HS6ST2 with prognosis, tumor immunity, and drug resistance
Source: Sci Rep. 2023 Nov 6;13:19209. doi: 10.1038/s41598-023-46525-x (PMC10628205; doi:10.1038/s41598-023-46525-x)
Supplement: Supplementary file 8 — Supplementary Legends. [file 41598_2023_46525_MOESM8_ESM.docx]

Table S1 Differential expression of HS6ST2 in pan-cancer.

Table S2 Association between HS6ST2 expression and DNA methylation in pan-cancer.

Table S3 Association between HS6ST2 expression and m6A, m5C, and m1A-related genes in pan-cancer.

Table S4 Association between HS6ST2 expression and mismatch repair (MMR) gene in pan-cancer.

Table S5 Association between HS6ST2 expression and microsatellite instability (MSI) in pan-cancer.

Table S6 Association between HS6ST2 expression and tumor mutational burden (TMB) in pan-cancer.

Table S7 Relationship Between HS6ST2 Expression and tumor microenvironment in pan-cancer.

Table S8 Relationship Between HS6ST2 Expression and Levels of Tumor Immune Cell Infiltration in pan-cancer.

Table S9 Relationship Between HS6ST2 Expression and Levels of Chemokines-Related Genes in pan-cancer.

Table S10 Relationship Between HS6ST2 Expression and Levels of Chemokines Receptor-Related Genes in pan-cancer.

Table S11 Relationship Between HS6ST2 Expression and Levels of MHC-Related Genes in pan-cancer.

Table S12 Relationship Between HS6ST2 Expression and Levels of Immunoinhibitory -Related Genes in pan-cancer.

Table S13 Relationship Between HS6ST2 Expression and Levels of Immunostimulator -Related Genes in pan-cancer.

Table S14 Relationship Between HS6ST2 Expression and Levels of Immune checkpoint inhibitors-Related Genes in pan-cancer.

Table S15 Relationship Between HS6ST2 Expression and Levels of Immune checkpoint stimulator-Related Genes in pan-cancer.

Table S16 The Prediction of the Correlation Between HS6ST2 Expression and Drug Sensitivity in pan-cancer.
